# Dynamics of nanoparticles in a 3D breathing lung-on-a-chip

**DOI:** 10.1007/s13346-025-01853-5

**Published:** 2025-04-16

**Authors:** Zohreh Sheidaei, Pooria Akbarzadeh, Navid Kashaninejad

**Affiliations:** 1 Faculty of Mechanical Engineering, Shahrood University of Technology, Shahrood, Iran; 2 Environmental Engineering Institute, School of Architecture, Civil and Environmental Engineering, Ecole Polytechnique Fédérale de Lausanne (EPFL), 1015 Lausanne, Switzerland; 3 Institute of Fluid Mechanics and Heat Transfer, Johannes Kepler University Linz, Linz, Austria; 4 Queensland Micro- and Nanotechnology Centre, Nathan Campus, Griffith University, 170 Kessels Road, Brisbane, QLD 4111 Australia

**Keywords:** Breathing lung-on-a-chip, Membrane stretch, Nanoparticle, Fluid–structure interaction, Numerical simulation

## Abstract

The"Breathing Lung-on-a-Chip,"a novel microfluidic device featuring a stretchable membrane, replicates the natural expansion and contraction of the human lung. It provides a more realistic in-vitro platform to study respiratory diseases, particle deposition, and drug delivery mechanisms. This device enables investigations into the effects of inhaled nanoparticles (NPs) on lung tissue and supports the development of advanced inhalation therapies. Uniform and optimal concentration delivery of NPs to cultured cells within the chip is critical, particularly as membrane stretching significantly influences particle dynamics. To address this, we developed a 3D numerical model that accurately simulates NP behavior under dynamic conditions, overcoming experimental limitations. The model, validated against experimental data, explores the effects of flow dynamics, particle size, membrane porosity, and stretching frequency/intensity on NP deposition in the air channel and transfer through the porous membrane into the medium channel. The results indicate that increased membrane stretch enhances the sedimentation rate of NPs in the air channel, thereby promoting their transfer to the medium channel, particularly in membranes with initially low porosity. Additionally, excessive stretching frequencies or intensities can introduce reverse flow and stagnation, leading to a longer residence time for NPs and altering their sedimentation patterns. These insights advance our understanding of NP transport in dynamic lung environments, paving the way for more effective applications of lung-on-a-chip technology in toxicological assessments and respiratory therapy innovations.

**Graphical Abstract:**

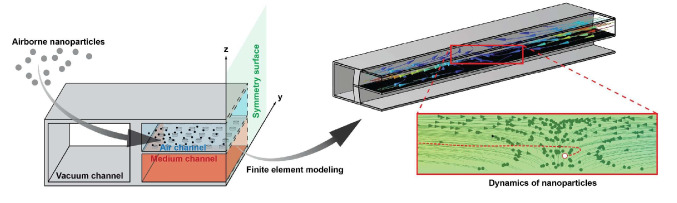

**Supplementary Information:**

The online version contains supplementary material available at 10.1007/s13346-025-01853-5.

## Introduction

**Fig. 1:**
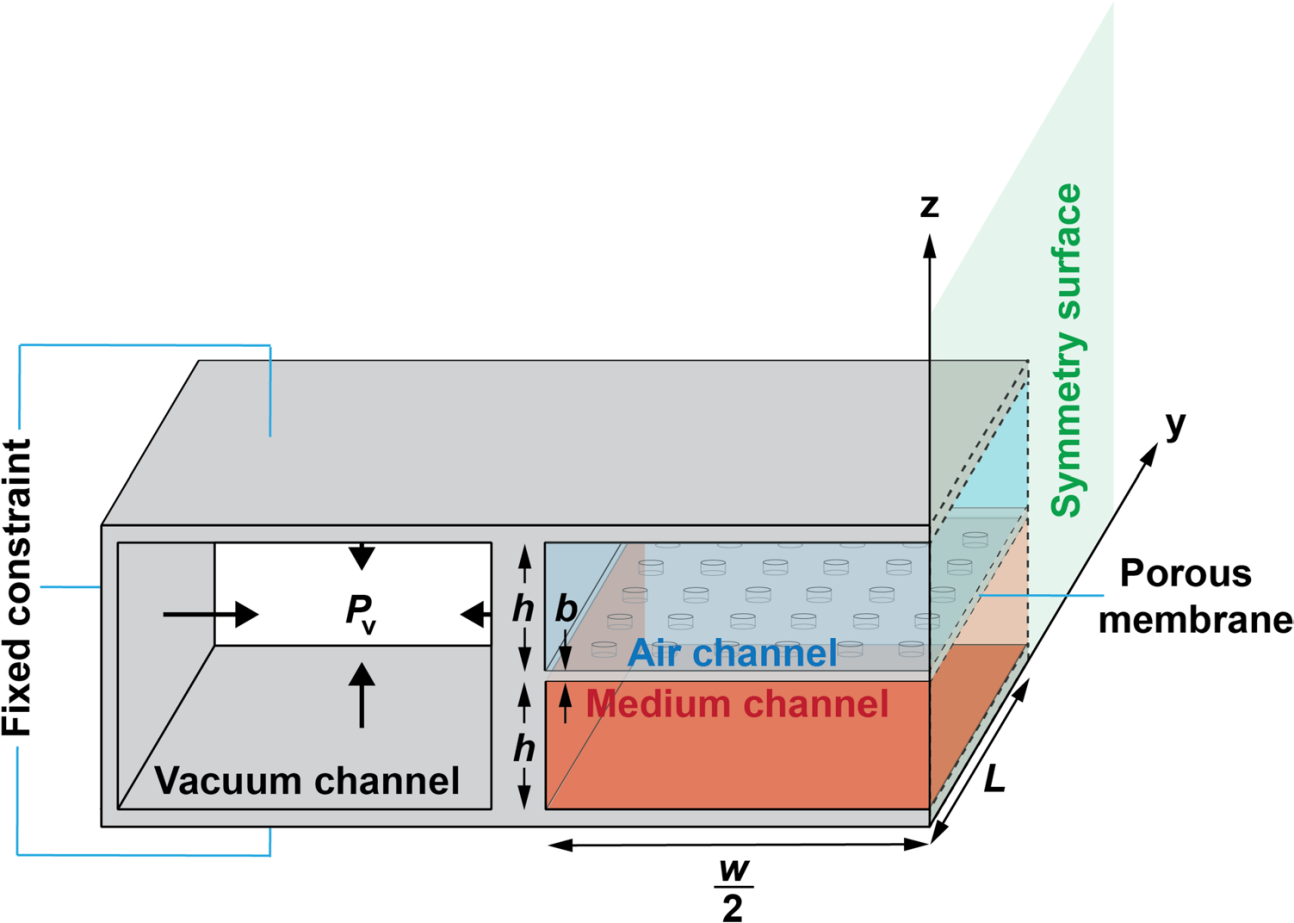
A schematic symmetrical lung-on-a-chip model containing a central air-medium dual channel separated by a porous membrane. Pulsatile pressure is applied to the lateral vacuum channels to provide the membrane’s breathing pattern and cyclical stretch

Continuous exposure to environmental pollutants through breathing is a known risk factor for severe pulmonary diseases and long-term health complications [1, 2]. Concurrently, the respiratory transportation of particles represents a promising method for developing non-invasive inhalation therapies, facilitating efficient drug delivery. The importance of these areas in biomedical research has led to an increasing focus on the behavior of particles within the human pulmonary system, particularly within toxicology and pharmaceutical studies [3–5]. Despite the progress, challenges remain in accurately delivering particles in in-vivo models, and the limitations of conventional in-vitro models to mimic realistic physiochemical conditions hinder further advancements in this research domain [6–8]. The development of lung-on-a-chip technology in 2010 offered a novel approach to address these challenges, realistically replicating the air-blood barrier of the lungs within a microchannel and allowing for direct exposure of cells to particle flows [9–11]. This system uses vacuum channels on both sides of the central cell channel, enabling the cyclic stretching of the membrane to mimic various breathing scenarios, as depicted in Fig. 1.

Today, this streamlined chip-based in-vitro approach has gained significant attention to explore new therapeutic and pathological processes [12–15]. To obtain accurate results with the lung-on-a-chip, it is crucial to regulate the delivery of studied particles to target cells at a consistently effective concentration [16, 17], as variability in their deposition fraction can result in differing toxic impacts on lung cells [18]. However, it should be considered that the diverse physical properties of particles, which lead to varied dynamic behaviors under different fluid flows and mechanical stimuli [6, 7], make precise and controlled delivery essential for each specific category of particles. In this regard, experimentally examining the dynamics of small particles inside the lung-on-a-chip presents several significant challenges, including the difficulty of monitoring and tracing particles through microchannels using transient microscopy, the tedious and repetitive process of controlling the delivery of various particles through microtubing and the high costs associated with the utilization of expensive particles.

Recently, numerical techniques have become a vital complement to experimental analyses in lung-on-a-chip devices, addressing their complex characteristics [19]. Moghadas et al. explored how airflow velocity affects NPs’ distribution in a 3D microchannel, finding that increasing fluid velocity from 0.1 mm/s to 0.612 mm/s aids in directing small, diffusion-dominant particles (diameter < 700 nm) toward the cell culture area [17]. Arefi et al. examined the roles of Brownian and gravity forces on NP sedimentation rates inside a 3D air channel showing that Brownian force crucially influences the deposition of NPs smaller than 500 nm [20]. Sheidaei et al.’s study in a 2D lung-on-a-chip model assessed NP dynamics across the air and medium channels separated by a porous membrane [21]. Their findings revealed that NPs larger than 500 nm distribute more uniformly at an airflow velocity of 1 mm/s in the air channel’s cell region than smaller particles. Additionally, smaller NPs (< 300 nm) tend to scatter throughout the media channel’s height, whereas larger particles are drawn closer to the cultured endothelial cells at the channel’s top wall.

Although these studies have explored the impact of key parameters on NP dynamics, they have focused on simplified reduced-order models of lung-on-a-chip, considering only its air channel or 2D model. However, the recognition that cells respond to mechanical stimuli in their environment, influencing critical biological processes such as tumorigenesis, phagocytosis, morphogenesis, and tissue repair [22–26], has underscored the importance of the lung-on-a-chip’s stretching feature. Therefore, a research gap remains in the comprehensive evaluation of NP dynamics using a realistic 3D model of the stretching lung-on-a-chip, which is essential for advancing pathological and therapeutic investigations of inhaled NPs on lung cells in a more physiologically relevant context. Applying vacuum pressure to stretch the membrane induces deflection of the air and medium channels’ side walls, potentially altering fluid hydrodynamics. Thus, alongside the particles’ physical properties and fluid inflow velocity, the cyclic stretching of the membrane emerges as a crucial factor potentially influencing fluid dynamics and, consequently, injected particles’ behavior within the lung-on-a-chip. Prior numerical studies [27, 28] have explored the impact of vacuum pressure and chip dimensions on membrane stretching. For instance, Huang and Nguyen’s 2D numerical analysis indicated that increasing membrane thickness and the distance between the vacuum and air-medium channels reduces membrane stretching [27]. While the detailed effects of membrane stretch on fluid-particle dynamics within the lung-on-a-chip device have still remained largely unexplored, studies have shown that the lung breathing pattern, characterized by stretch frequency and intensity, plays a crucial role as a key determinant in the transport and deposition of aerosols within the human respiratory tract [29].

To address the knowledge gaps, our study sets out to create a comprehensive 3D computational model of a real lung-on-a-chip that integrates solid deformation, fluid flow, and particle tracing physics.

This model allows to meticulously examine how porous membrane stretching influences fluid velocity and, consequently, the dynamics of the injected NPs inside the microchannels.

The obtained findings on deposit and transfer rates of particles with varying diameters under different physiological cyclic breathing conditions are expected to effectively contribute in the precise adjustment of particle dosages and chemical formulations, thereby enhancing the delivery of optimal particle concentrations to target cells. This, in return, will ultimately improve toxico-pharmacokinetic outcomes derived from the lung-on-chip technology.

This paper is structured as follows: The Problem definition section outlines the problem, offering a clear definition to guide the reader. The Numerical modeling section delves into the numerical modeling aspects, including governing equations, boundary conditions, initial values, and details of the meshing procedure. The Model verification section validates the numerical results by comparing them with previously published data and an experimental study, ensuring the model’s reliability. In the Results and discussions section, the results are thoroughly analyzed. Lastly, the Conclusion section summarizes the conclusions and provides an overview of the findings, highlighting the significance of the research and potential future directions.

## Problem definition

This study’s principal aim is to utilize a numerical model for an in-depth analysis of NP dynamics within the dual gas–liquid channel of a lung-on-a-chip, influenced by cyclic stretching that simulates breathing patterns. It investigates how particle size, flow rate, membrane porosity, and stretching frequency and intensity affect NP distribution within the lung-on-chip microenvironment. A time-dependent 3D finite element model is crafted using the “Fluid–Structure Interaction” and “Particle Tracing for Fluid Flow” interfaces in COMSOL Multiphysics software, inspired by the design introduced by Huh et al. [30], albeit with a modification to the membrane pores’ shape from circular to regular octagonal. This adjustment aims to address computational convergence issues while maintaining result accuracy, leveraging the geometric benefits of octagons to approximate circles.

Focusing on fluid-driven particle dynamics, this research deliberately excludes cellular interactions, concentrating instead on a bare lung-on-chip model to simplify the fluid–solid interaction problem. It assumes only the impact of structural displacement on fluid domain deformation, omitting the fluid’s reciprocal influence on the solid structure due to low fluid velocity and pressure gradient. The methodology unfolds in three stages: analyzing structural deformation due to vacuum stretching, examining fluid behavior within the deformed microchannel geometry, and investigating NP dynamics under stimulated fluid flows in these channels.

Considering the literature that particles smaller than 1 µm can reach the lung alveolus [31], this study models NPs ranging from 10 to 900 nm. To mimic actual respiratory rhythms, the membrane is subjected to vacuum-induced sinusoidal stretches at excitation frequencies of 0.25 Hz and 1 Hz, with strain levels of 10%, 15%, and 20% [32]. Key simulation inputs are summarized in Table 1, setting the stage for a comprehensive exploration of NP behavior in a dynamically stretching lung-on-a-chip environment.

**Table 1:** Important input values that were used in the simulations of the present study

Parameters	Values
Height of the channels [27, 33]	100 [µm]
Length of the channels [20]	2 [mm]
Width of the channels [27]	400 [µm]
Thickness of the membrane [27]	10 [µm]
Density of particles [20]	1180 [kg/m^3^]
Density of media [34]	1000 [kg/m^3^]
Viscosity of media [34]	0.718 [mPa.s]
Density of air [35]	1.123 [kg/m^3^]
Viscosity of air [35]	0.019 [mPa.s]
Medium flow rate at the inlet [21]	0.72 [µL/min], 24 [µL/min]
Air flow rate at the inlet [21]	0.72 [µL/min], 24 [µL/min]
Young’s modulus of solid [36]	1.22 [MPa]
Poisson’s ration [27]	0.49
Density of solid [36]	996 [kg/m^3^]
Membrane strain[32]	10%, 15%, 20%
Frequency of vacuum pressure[32]	0.25 [Hz], 1 [Hz]

## Numerical modeling

This section explores the governing equations, forces on NPs, and the initial and boundary conditions shaping our computational study. The domain under consideration contains two distinct fluids, air, and medium, both governed by the same set of equations. A unified general equation is presented to streamline the discussion, although it is crucial to factor in the unique physical properties of each fluid within these equations. The differentiation in fluid properties is essential for accurate simulation results. Towards the end of this section, the meshing procedure, an integral part of setting up the computational model, is also briefly outlined. This step ensures that the computational grid is adequately fine-tuned to capture the complex dynamics of fluid flow and NP movement within the lung-on-a-chip environment.

### Governing equations

Considering elastic and compressible structural behavior, membrane stretch and the consequent deformation of the microchannels are solved by Newton’s second law of motion [37]:

1 $${\rho }_{s}\frac{{\partial }^{2}{u}_{s}}{{\partial t}^{s}}=\nabla .(FS)$$

Here, ${\rho }_{\text{s}}$ is the solid mass density, ${{\varvec{u}}}_{{\varvec{s}}}$ is the displacement, and ${\varvec{F}}={\varvec{I}}+\nabla {u}_{s}$ is the deformation gradient in which ${\varvec{I}}$ is the identity matrix. ${\varvec{S}}$ is the Second Piola–Kirchhoff stress tensor, which calculates forces in the deformed solid domain concerning its reference configuration:

2 $$\mathbf{S}=\frac{{\nu }_{\text{s}}{E}_{\text{s}}}{\left(1+{\nu }_{\text{s}}\right)\left(1-2{\nu }_{\text{s}}\right)}{\mathbf{E}}^{T}\mathbf{I}+\frac{{E}_{\text{s}}}{\left(1+{\nu }_{\text{s}}\right)}\mathbf{\rm E}$$

in which $\boldsymbol{\rm E}=\left({{\varvec{F}}}^{T}{\varvec{F}}-{\varvec{I}}\right)/2$ is Green–Lagrange strain tensor. ${E}_{\text{s}}$ and ${\nu }_{\text{s}}$ are material Young’s modulus and Poisson’s ratio, respectively.

Accordingly, the interaction between the fluids and the deforming solid structure is modeled using the Arbitrary Lagrangian–Eulerian (ALE) method, where a modified form of the Navier–Stokes equation describes the fluid motion as follows [38]:

3 $$\nabla \bullet {\varvec{v}}=0$$

4 $$\rho \left(\frac{\partial {\varvec{v}}}{\partial t}+{{\varvec{v}}}_{c}\bullet \nabla {\varvec{v}}\right)=\nabla \bullet{\varvec{\sigma}}$$

where ${\varvec{v}}$ and $\rho$ are fluid velocity and mass density, respectively. Similar to the medium flow, the airflow is characterized as an incompressible and continuous fluid [21]. ${{\varvec{v}}}_{c}$ is the relative convective velocity between the fluid velocity and the velocity of mesh-node $\left({{\varvec{v}}}_{\text{c}}={\varvec{v}}-{{\varvec{v}}}_{\text{n}}\right)$. ${\varvec{\sigma}}$ denotes Cauchy stress tensor, which for an incompressible and Newtonian fluid is equal to:

5 $${\varvec{\sigma}}=-p{\varvec{I}}+\mu \left(\nabla {\varvec{v}}+\nabla {{\varvec{v}}}^{T}\right)$$

where $p$ is the fluid pressure field, and $\mu$ is the fluid dynamic viscosity. The ALE technique manages the dynamics of the deforming domain by enabling mesh motion. Grid deformation and velocity of the node at each station are predicted by a mesh-update procedure based on the movement of the boundaries and mesh smoothing. In this study, the “Yeoh” method is chosen for mesh smoothing because it can handle large deformations by effectively preventing mesh entanglement and inversion [39]. For a comprehensive understanding of the ALE technique and mesh-update procedure, one can refer to references [37, 38]. The solid and fluid domains are coupled through the following conditions established at the fluid–structure interaction interface:

6 $${\varvec{v}}={\dot{{\varvec{u}}}}_{s}$$

7 $${\varvec{\sigma}}\bullet {\varvec{n}}={{\varvec{\sigma}}}_{s}\bullet {\varvec{n}}$$

${{\varvec{\sigma}}}_{s}$ is the solid Cauchy stress tensor which is derived from the deformation gradient:

8 $${{\varvec{\sigma}}}_{s}=\frac{1}{\left|{\varvec{F}}\right|}{\varvec{F}}{\varvec{S}}{{\varvec{F}}}^{T}$$

The motion of particles within the fluid channels of the chip are modeled using the Lagrangian approach as follows, which enables the discrete tracking of the particles’ trajectories [17, 20, 21]:

9 $${m}_{p}\frac{d{{\varvec{v}}}_{p}}{dt}=3\pi \mu \left({\varvec{v}}-{{\varvec{v}}}_{p}\right){d}_{p}+{{\varvec{F}}}_{b}+{m}_{p}\left(1-\frac{\rho }{{\rho }_{p}}\right)\mathbf{g}$$

where ${m}_{\text{p}}$, ${d}_{\text{p}}$, and ${\rho }_{\text{p}}$ are respectively the particle mass, diameter, and density. ${{\varvec{v}}}_{p}$ indicates the particle velocity in air and media channels. The right-hand side of Eq. 9 is the summation of the drag force, the Brownian force, and the particle’s buoyant weight. The Brownian force (${{\varvec{F}}}_{b}$) in Eq. 9 is determined as [40]:

10 $${{\varvec{F}}}_{b}={\varvec{\zeta}}\sqrt{6\pi {k}_{B}\mu T{d}_{p}/\Delta t}$$

where ${T}_{i}$ represents the fluid temperature, which is considered to be at a constant level of $37^\circ \text{C}$, and $\Delta t$ is the time step. The vector ${\varvec{\zeta}}$, whose components are randomly selected with a Gaussian distribution, demonstrates the direction of Brownian force.

### Boundary conditions, initial values, and meshing procedure

To optimize computational resources without compromising the accuracy of our simulations, we analyze a symmetrical lung-on-a-chip model (see Fig. 1). Dynamic stretching of the porous membrane is achieved by applying pulsatile vacuum pressure to the vacuum channel walls. All external surfaces of the chip are fixed except those perpendicular to fluid flow. These are designated as"Roller"boundaries to restrict displacement in the normal direction. We apply fully developed flow rates and zero-pressure boundaries at the entrances and exits of both air and medium channels. Initial fluid velocity and solid displacement values are set to zero. A"No-slip"condition is enforced at all solid–fluid interfaces within the channels. NPs, numbered at 2000, are introduced at the air channel’s entrance. Their airflow velocity is matched, and their initial positions are randomly assigned at the inlet boundary. The air channel substrate acts as a sticky boundary, capturing airborne NPs to evaluate their deposition efficiency. The remaining side walls of the fluid channels implement a"Bounce"boundary condition. The shared surface between the air and medium channels is considered permeable, allowing for a"Pass through"boundary condition that permits NPs to move freely into the lower channel.

In our meshing strategy, we employ an array of regular octagonal hollows in the membrane instead of actual circular shapes. This decision addressed convergence issues associated with mesh quality in our numerical simulations. Mesh quality is critical for accurately capturing the modeling geometry, particularly for structures with curved boundaries. To nearly represent the arc of each circular surface of the pore, a minimum of 40 elements is required. This requirement introduces a significant degree of freedom due to the large number of pores along the membrane. Conversely, reducing the number of mesh elements leads to a suboptimal representation of the circular domain, adversely affecting the accuracy of the fluid results and, consequently, the behavior of NPs around the pores on the air channel substrate. In scenarios with a coarse mesh, it was observed that particles disappear from the computational domain due to a lack of fluidic data in unmapped regions of the pores. In contrast, a regular octagonal surface can be effectively modeled with a minimum of eight triangular elements. Thus, regular octagonal pores, which have an area comparable to circular pores, significantly expedite the simulation convergence. The computational domain of the lung-on-a-chip is discretized into $1.2\times {10}^{6}$ elements.

### Model verification

**Fig. 2:**
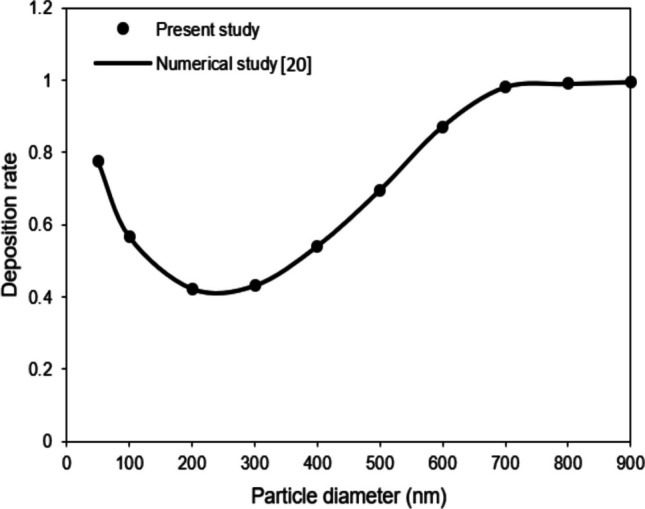
Contrasting the outcomes of the current study with the numerical analysis presented in Ref. [20] regarding the deposition rate of airborne NPs with different diameters in a microchannel while considering an inlet airflow velocity of 0.337 mm/s at the entrance

In this section, the accuracy of the current numerical model is evaluated through a comparative approach, using both literature data and experimental results for reference. Initially, a 3D limiting-case verification is conducted by setting membrane porosity to zero, aiming to observe the dynamics of NPs within a microchannel. NPs, with diameters ranging from 10 to 900 nm, are propelled by airflow through the channel with dimensions of 400 µm × 70 µm × 2000 µm. This model calculates the deposition rate of particles on the channel substrate, defined as the ratio of settled to released particles, and compares these calculations with the 3D numerical findings of Arefi et al. [20]. This comparison considers all relevant physical and parametric values and assumptions about boundary conditions, as detailed in Ref. [20]. The results, illustrated in Fig. 2, show that the deposition rate of NPs significantly depends on their diameter at a consistent inlet air velocity of 0.337 mm/s, aligning closely with the observations made by Arefi et al. [20] (${R}^{2}=1$).

To further validate the accuracy of the current numerical model, an experimental study was conducted focusing on particle deposition. Particles measuring 1 µm and 10 µm in diameter were tested in a microchannel under the water flow rates of 9 µL/min and 27 µL/min, respectively. The particles had 2200 kg/m^3 densities for the smaller ones and 1470 kg/m^3 for the larger ones. The experimental setup for the particle injection is explained in Section 1 of the Supplementary Information.

**Fig. 3:**
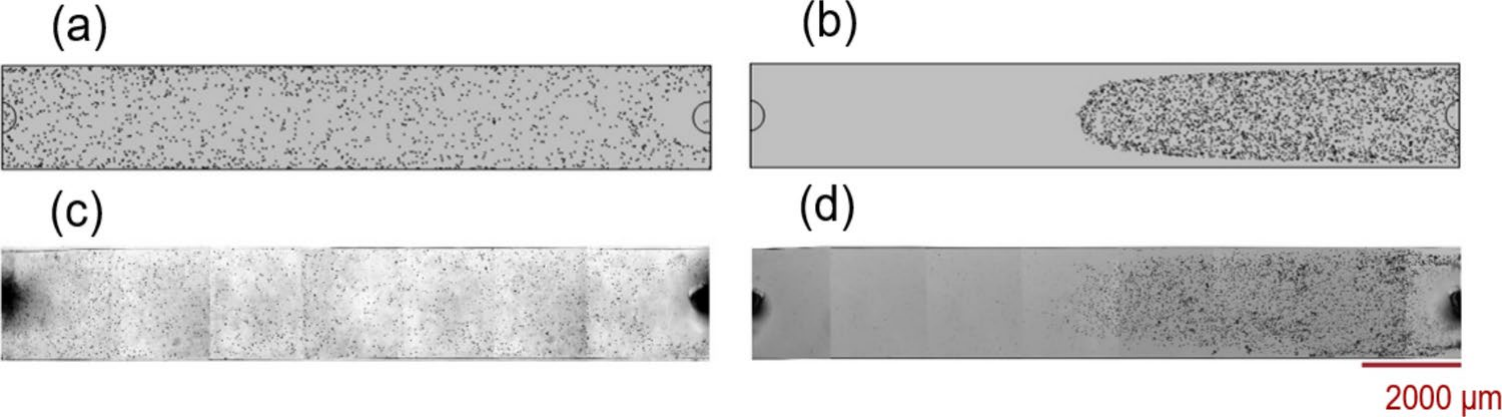
Numerical simulation of particles’ distribution with a diameter of **a** 1 μm and **b** 10 μm on the substrate of a microchannel, respectively, under the water flow of 9 µL/min and 27 µL/min. The corresponding experimental verification of the numerical results for the particles of **c** 1 μm and **d** 10 μm

Post-injection, images of the deposited particles on the substrate were captured (as seen in Fig. 3c and d). The 3D numerical model was carefully aligned with the experimental geometry to ensure the consistency of numerical and experimental results. Figure 3a and c highlight the successful uniform distribution of 1 µm particles at a 9 µL/min flow rate. Conversely, the 27 µL/min flow rate was less effective in moving the larger 10 µm particles throughout the microchannel, as shown in Fig. 3b and d. Notably, this validation primarily aimed to examine the settlement trajectory patterns of particles on the channel substrate, while fluid–structure interactions were neglected, as they predominantly influence the particle deposition rate, which remains challenging to measure accurately through experimental techniques.

The lung-on-a-chip device is typically made from Polydimethylsiloxane (PDMS) due to its flexibility and robustness during cyclic stretching. However, it is crucial to note that PDMS’s elastic properties can vary based on fabrication and coating materials [41]. Achieving an accurate and realistic measure of the device’s elasticity is vital for the precision of subsequent numerical simulations. This necessitates a comparative analysis of membrane mechanical strain against vacuum pressure, comparing experimental data from a lung-on-a-chip device with a corresponding numerical model. To ensure accuracy, the geometry of the primary numerical model was adjusted to align with the device as outlined in Ref. [42], assuming a uniform elastic modulus across the microchip components.

**Fig. 4:**
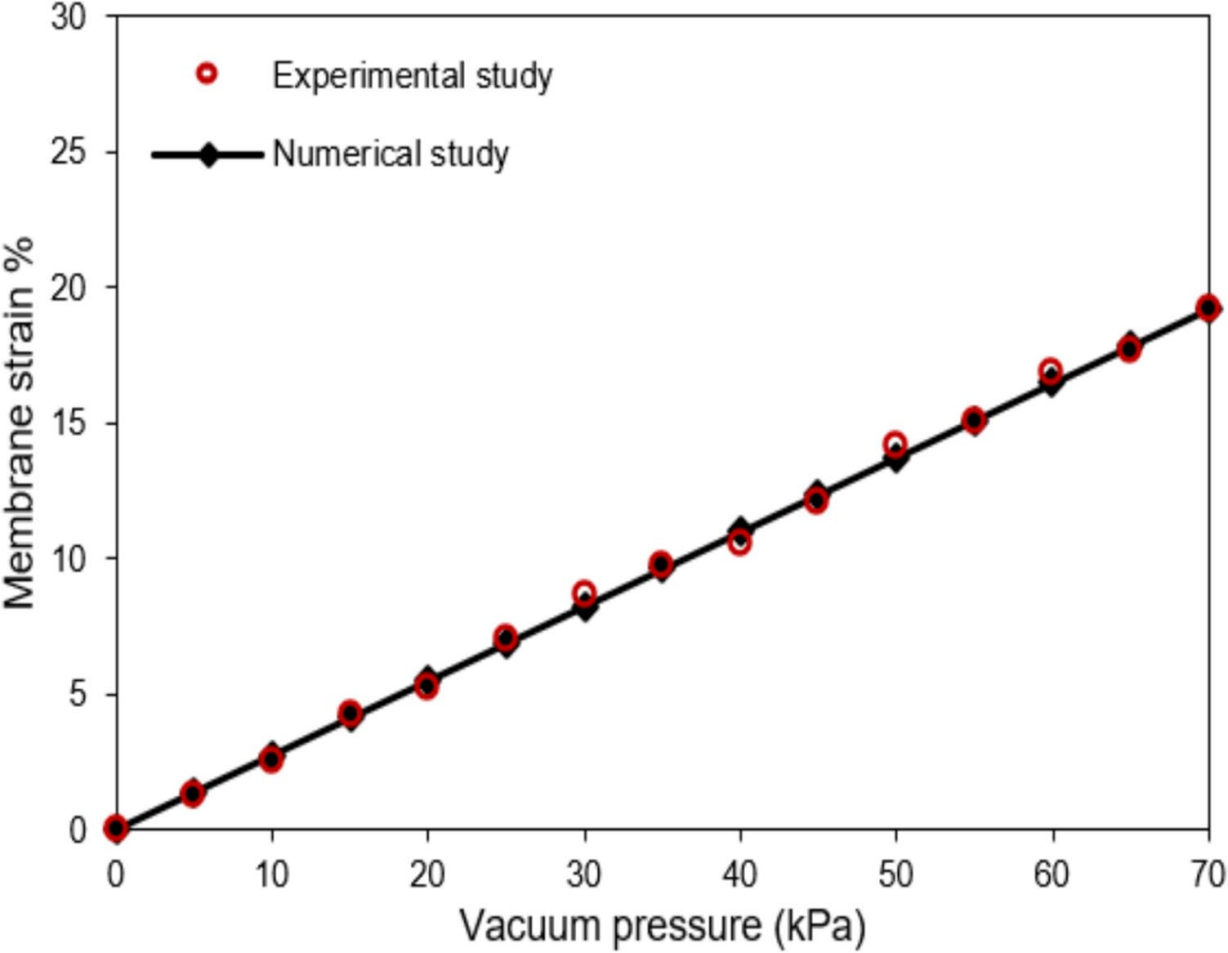
Comparison of the present numerical results with an experimental analysis for the strain of the porous membrane under different vacuum pressures

Membrane strain measurements were taken under varying vacuum pressures, and these measurements were compared to the numerical model’s predictions. Young’s modulus for the microchip was estimated through iterative adjustments and employing the least mean squares method. As shown in Fig. 4, a high degree of agreement was found, with Young’s modulus determined to be 1.31 MPa (${R}^{2}=0.99$). This value aligns closely with the previously reported elasticity of PDMS, 1.22 MPa [36], underscoring the model’s accuracy in reflecting the material’s properties.

## Results and discussions

This section presents the numerical results from a 3D multi-physics simulation, evaluating the effects of key parameters—fluid flow rate, membrane porosity, and the intensity and frequency of membrane stretching—on the dynamics of NPs across different size ranges in a lung-on-a-chip. To better demonstrate the importance of such a comprehensive 3D model of a stretching lung-on-a-chip in accurately evaluating particle dynamics, the results are sequentially presented, covering: dynamics of nanoparticles in a fixed stretched lung-on-a-chip; fluid dynamics inside a sinusoidal stretched lung-on-a-chip; and Dynamics of nanoparticles in a sinusoidal stretched lung-on-a-chip. To highlight the need for such a 3D modeling, a comparative study was conducted at the outset between the current 3D simulation with an earlier 2D model [21] to assess the strengths and limitations of the 2D approach. The results are presented in Supplementary Section 2.

### Dynamics of nanoparticles in a fixed stretched lung-on-a-chip

**Fig. 5:**
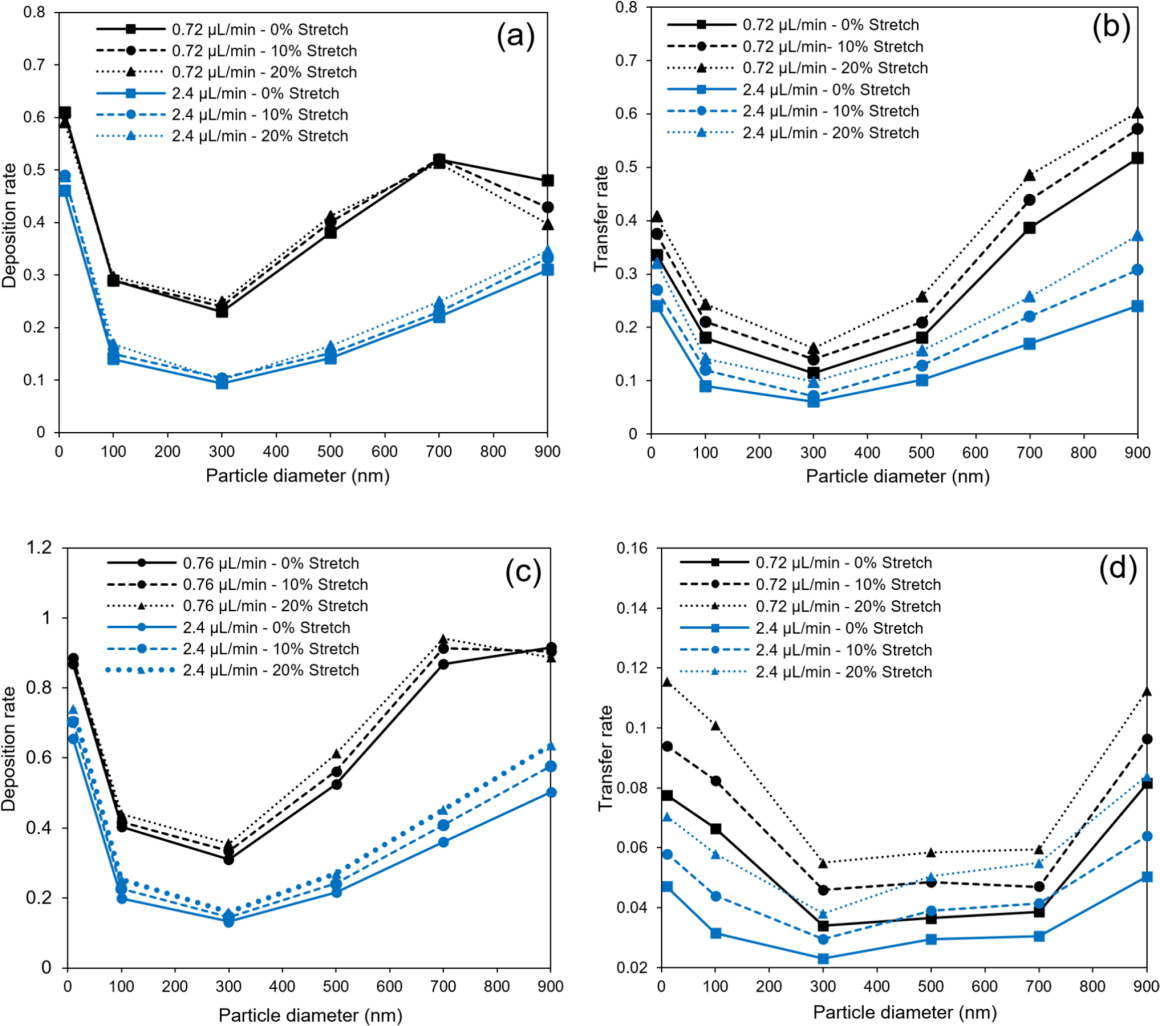
**a**) Deposition and **b**) transfer rates of NPs with various diameters under different stretching of 30.5% porous membrane and two different inflow rates. **c**) Deposition and **d**) transfer rates of NPs with various diameters under different stretching of 3.6% porous membrane and two different inflow rates

Before examining the dynamic effects of stretching, it is insightful to first assess the impact of stretching intensity on NP deposition and transfer rates under stationary conditions. To this end, two distinct membrane porosities of 3.6% and 30.5% are analyzed [11, 21]. These porosity values were selected based on prior experimental studies and represent two physiologically relevant regimes: a low-porosity membrane (3.6%) mimicking tighter epithelial barriers, and a high-porosity membrane (30.5%) designed for enhanced exchange between air and media channels. We also evaluate two different inflow rates: 0.72 μL/min and 2.4 μL/min. The influence of stretching intensity is illustrated with results for three scenarios, as shown in Fig. 5: No Stretching, 10% Membrane Strain, and 20% Membrane Strain. Stretching the membrane not only expands the cross-sectional area of the microchannel but also alters the porosity ratio of the membrane, as detailed in Table 2. The results clearly show that stretching the membrane increases its porosity, with particularly significant changes at lower porosity levels.

**Table 2:** Effect of stretching on membrane size, porosity area, and porosity ratio

	No Stretch		10% Strain		20% Strain	
	Low porosity (3.6%)	High porosity (30.5%)	Low porosity (3.6%)	High porosity (30.5%)	Low porosity (3.6%)	High porosity (30.5%)
Membrane size	8.0e5 μm^2^	8.0e5 μm^2^	8.8e5 μm^2^	8.8e5 μm^2^	9.6e5 μm^2^	9.6e5 μm^2^
Porous area	0.29e5 μm^2^	2.44e5 μm^2^	0.38e5 μm^2^	2.93e5 μm^2^	0.47e5 μm^2^	3.42e5 μm^2^
Porosity	3.6%	30.5%	4.3%	33.3%	4.9%	35.6%

When considering consistent inflow rates, the expansion of the microchannel inlet cross-section leads to reduced inflow velocities, which in turn increases the sedimentation rate. It is important to note that the sedimentation rate is directly proportional to the level of stretching but remains independent of the membrane porosity. This relationship is clearly demonstrated by identical outcomes in the summation of transfer and deposition rates for different porosities, as shown in Fig. 5.

The increased sedimentation rate, coupled with a higher porosity level caused by stretching, subsequently enhances the transfer rate, as depicted in Fig. 5b and d. The authors have observed that the transfer rate under stretching (${T}_{\text{s}}$) can be approximated by:

11 $${T}_{\text{s}}\cong {T}_{\text{u}}{R}_{1}{R}_{2}$$

where ${T}_{\text{u}}$ is unstretched transfer rate. ${R}_{1}$ signifies the ratio of sedimentation rates between the stretched and unstretched scenarios, obtained straightforwardly by summing the transfer and deposition rates. ${R}_{2}$ shows the porosity ratio, calculated by dividing the stretched porosity by the unstretched porosity from Table 2. The validity of Eq. 11 can be exemplified, for instance with 500 nm particles at the inflow rate of 0.72 μL/min under 10% strain of the high-porosity membrane, where ${T}_{\text{s}}=0.2095$, ${T}_{\text{u}}=0.18$, ${R}_{1}\cong 1.09$, ${R}_{2}\cong 1.09$. In addition, the deposition rate also demonstrates an increase with the stretching of the membrane, as depicted in Fig. 5a and c. However, it’s noteworthy that for NPs with a high sedimentation rate, stretching the membrane primarily results in an augmented transfer rate with a minimal impact on the sedimentation rate that eventually leads to consistent or decreased deposition rates.

### Fluid dynamics inside a sinusoidal stretched lung-on-a-chip

Before delving into the behavior of NPs, it is crucial to first understand the fluid dynamics within the microdevice under variable stretching conditions due to their strong influence on particles’ movement. In addition to the flow rate and average fluid velocity presented herein, the average pressure gradient of the fluid throughout a stretching cycle period is evaluated and detailed in Supplementary Section 3.

**Fig. 6:**
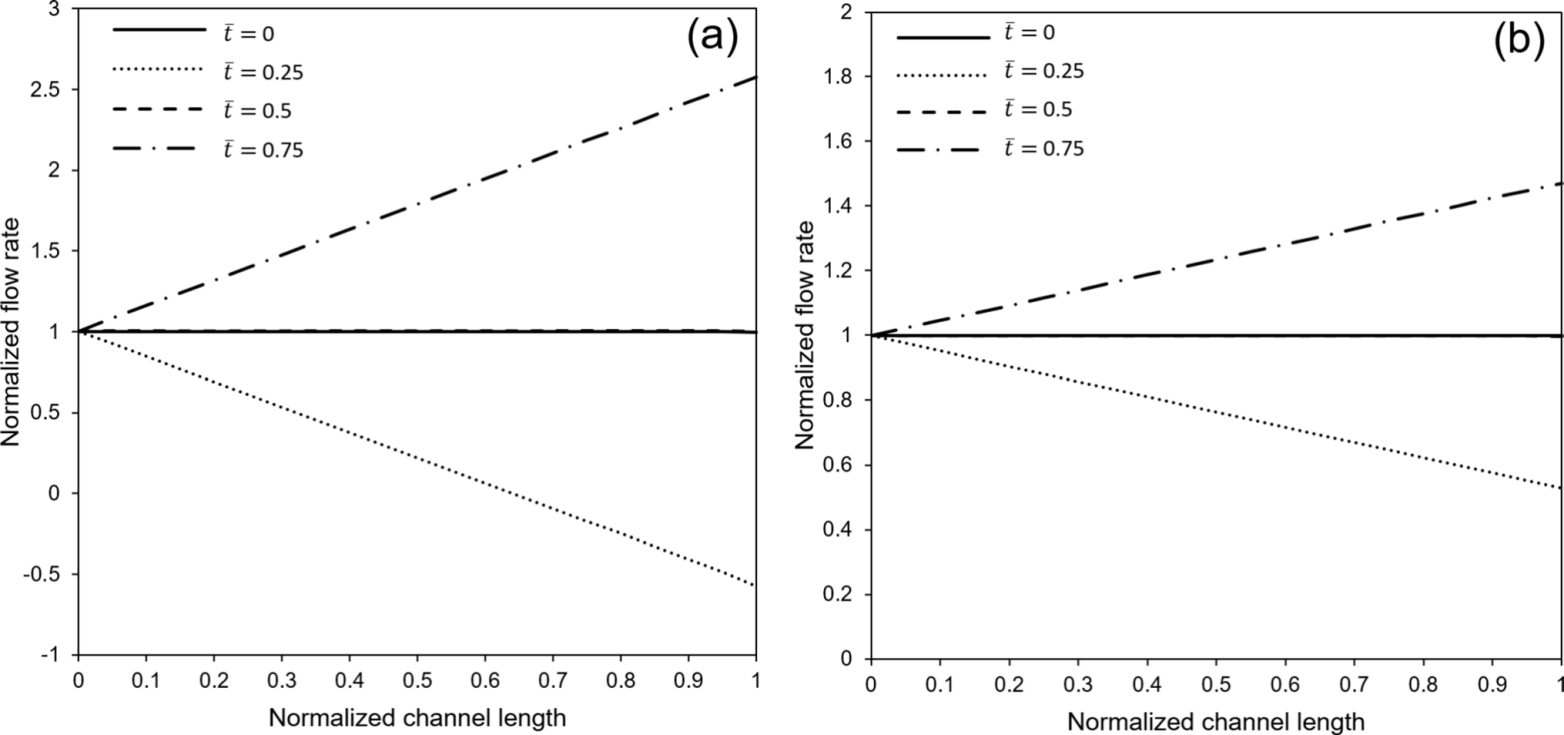
Normalized airflow rate along the normalized channel length in different times under sinusoidal membrane stretch and inflow rates of **a**) and **b**) 2.4 μL/min. The frequency and stretch of the membrane respectively equal to 1 Hz and 10%. Flow rate and distance are respectively normalized with respect to the inflow rate and channel length, while time is normalized with respect to the stretching cycle period

For our experiments, the membrane undergoes vacuum-induced sinusoidal stretches at frequencies of 0.25 Hz and 1 Hz, with strain levels of 10%, 15%, and 20% to mimic different breathing patterns. In this section, we focus our analysis on the membrane with a high porosity of 30.5% to clarify the principal observations related to airflow dynamics. Figure 6 shows the normalized flow rate along the channel at various normalized times. Here, flow rate and distance are normalized with respect to the inflow rate and channel length, respectively, while time is normalized concerning the stretching cycle period. The data represents a stretching cycle at an excitation frequency of 1 Hz.

Two distinct inflow rates are considered, and the vacuum level is adjusted to achieve a 10% strain at maximum membrane extension. According to the continuity equation, the flow rate difference between the inlet and outlet of the microchannel is inversely related to the time derivative of the control volume’s size. Notably, at $\overline{t }=0$ and $\overline{t }=0.5$, when the time derivative of the air channel volume is zero, the flow rate remains consistent along the channel length, as illustrated in Fig. 6.

However, during times of membrane expansion and compression at $\overline{t }=0.25$ and $\overline{t }=0.75$ —when the time derivative reaches its peak magnitude—the outlet flow rate becomes respectively lower and higher than the inlet. A particularly interesting phenomenon observed in Fig. 6, especially at lower inflow rates, relates to the continuity equation: when the time derivative of the volume exceeds the inflow rate, the excess must be accommodated through the outlet, resulting in a reversed flow and temporary local stagnations within the control volume.

**Fig. 7:**
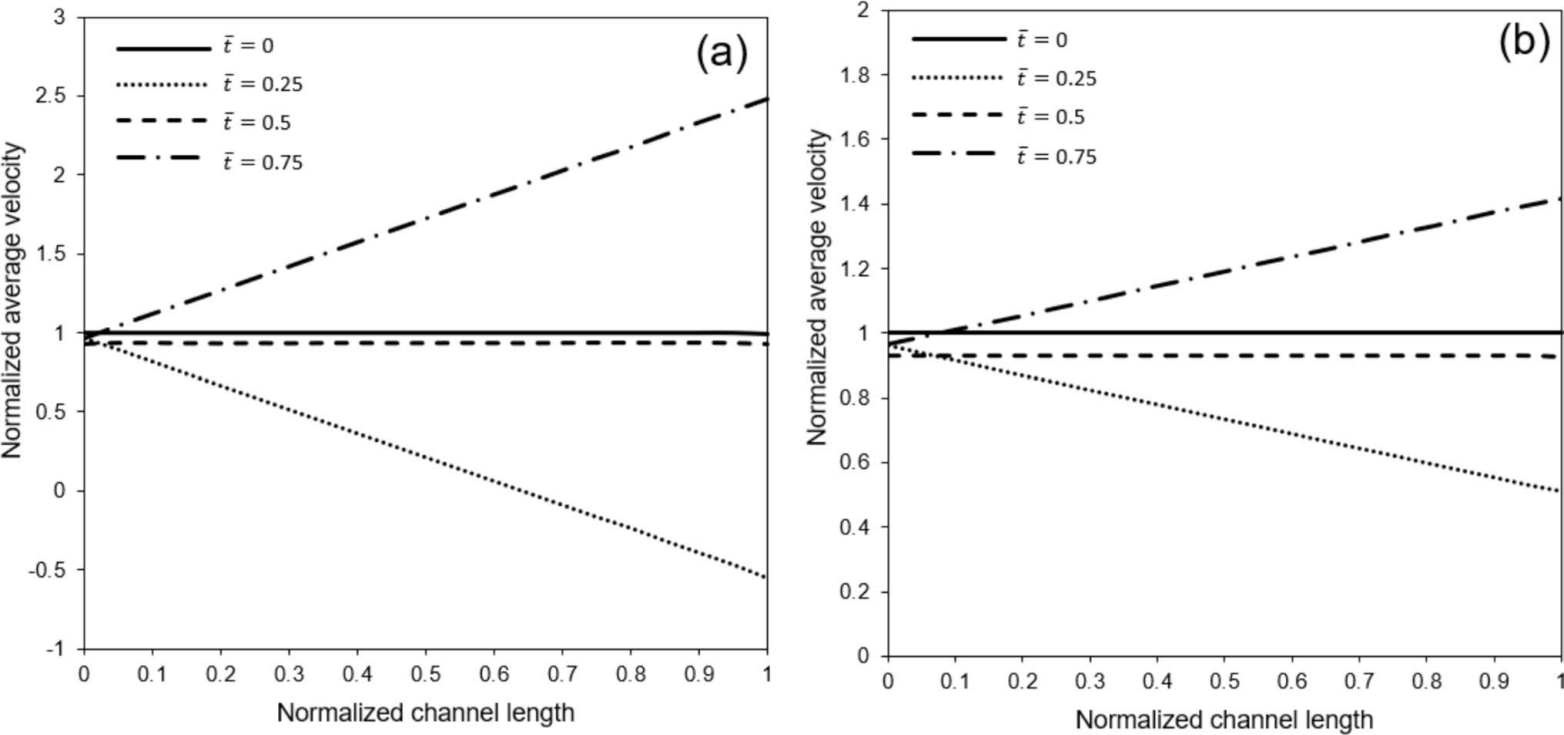
Normalized average fluid velocity along the channel length at different times under sinusoidal membrane stretch and inflow rates of **a**) 0.72 μL/min and **b**) 2.4 μL/min. The frequency and strain of the membrane respectively equal to 1 Hz and 10%. Average fluid velocity and distance are respectively normalized with respect to the mean inlet velocity and channel length, while time is normalized with respect to the stretching cycle period

Fig. 7 presents the normalized average fluid velocity along the channel at different times, where the fluid velocity is normalized concerning the mean velocity at the unstretched microchip inlet. The most noticeable observation in Fig. 7 is the impact of the air channel’s cross-sectional changes. Despite the flow rate’s consistent nature along the channel length, as illustrated in Fig. 6, the expansion of the control volume at $\overline{t }=0.5$ results in a drop in average flow velocity, where the normalized average velocity magnitude is slightly below 1. This effect of the cross-sectional increase also becomes apparent at $\overline{t }=0.25$ and $\overline{t }=0.75$, as depicted in Fig. 7.

**Fig. 8:**
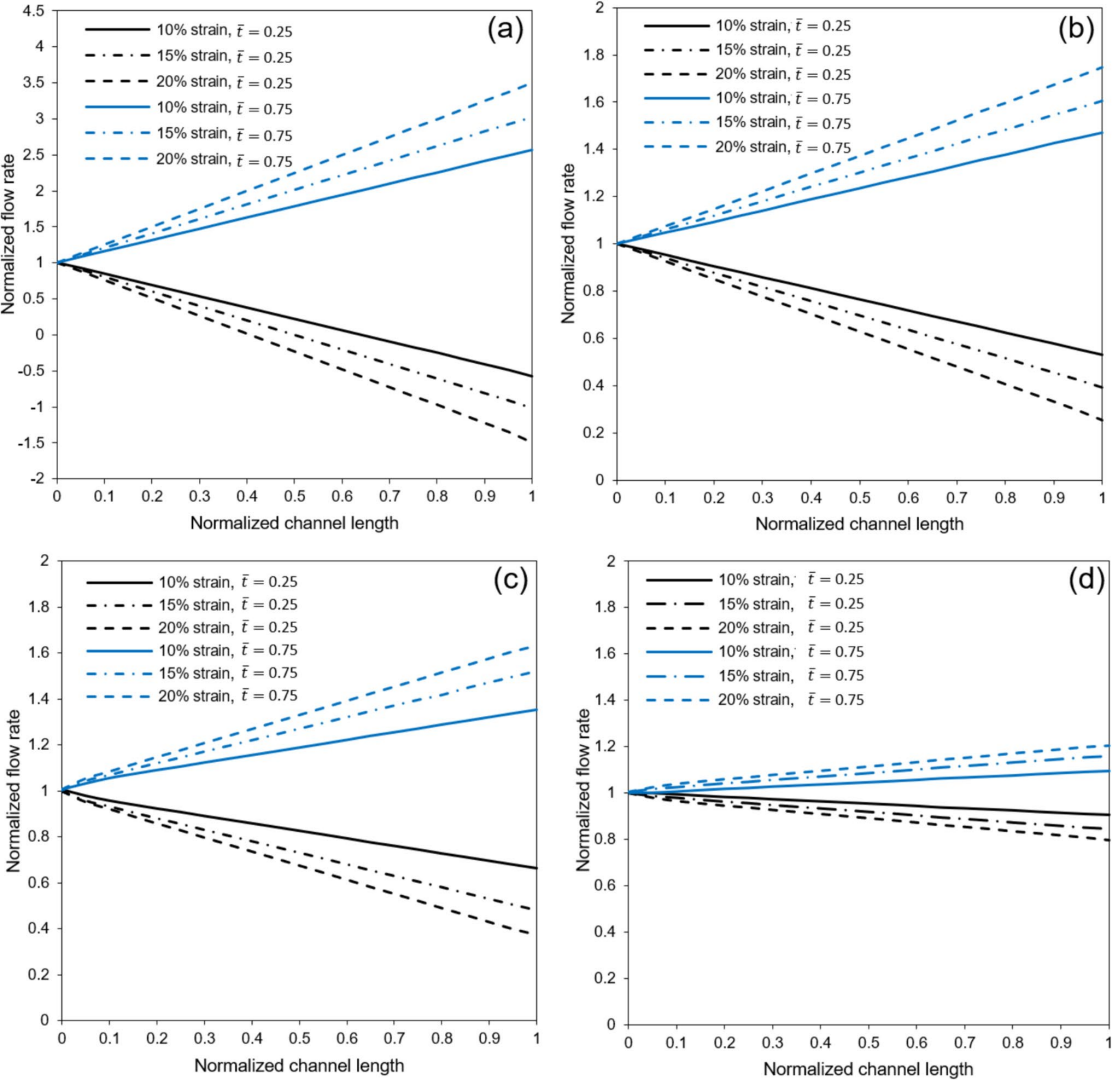
Normalized flow rate along the air channel under different membrane stretching intensities at two different times when **a**) stretching frequency is 1 Hz and inflow rate is 0.72 μL/min **b**) stretching frequency is 1 Hz and inflow rate is 2.4 μL/min, **c**) stretching frequency is 0.25 Hz and inflow rate is, and **d**) stretching frequency is 0.25 Hz and inflow rate is 2.4 μL/min. Flow rate and distance are normalized with respect to the inflow rate and channel length, respectively, while time is normalized concerning the stretching cycle period

Fig. 8 examines the impact of excitation frequency as well as stretching intensity on the normalized flow rate along the channel length at $\overline{t }=0.25$ and, when the time derivative of the control volume size reaches its peak magnitude. The findings suggest that augmenting the stretching intensity and frequency leads to a noticeable increase in the disparity between the flow rates at the inlet and outlet of the air channel. In addition, it can be concluded from Fig. 8a that the stagnation region moves closer towards the channel’s inlet by increasing stretching intensity at a given time.

**Fig. 9:**
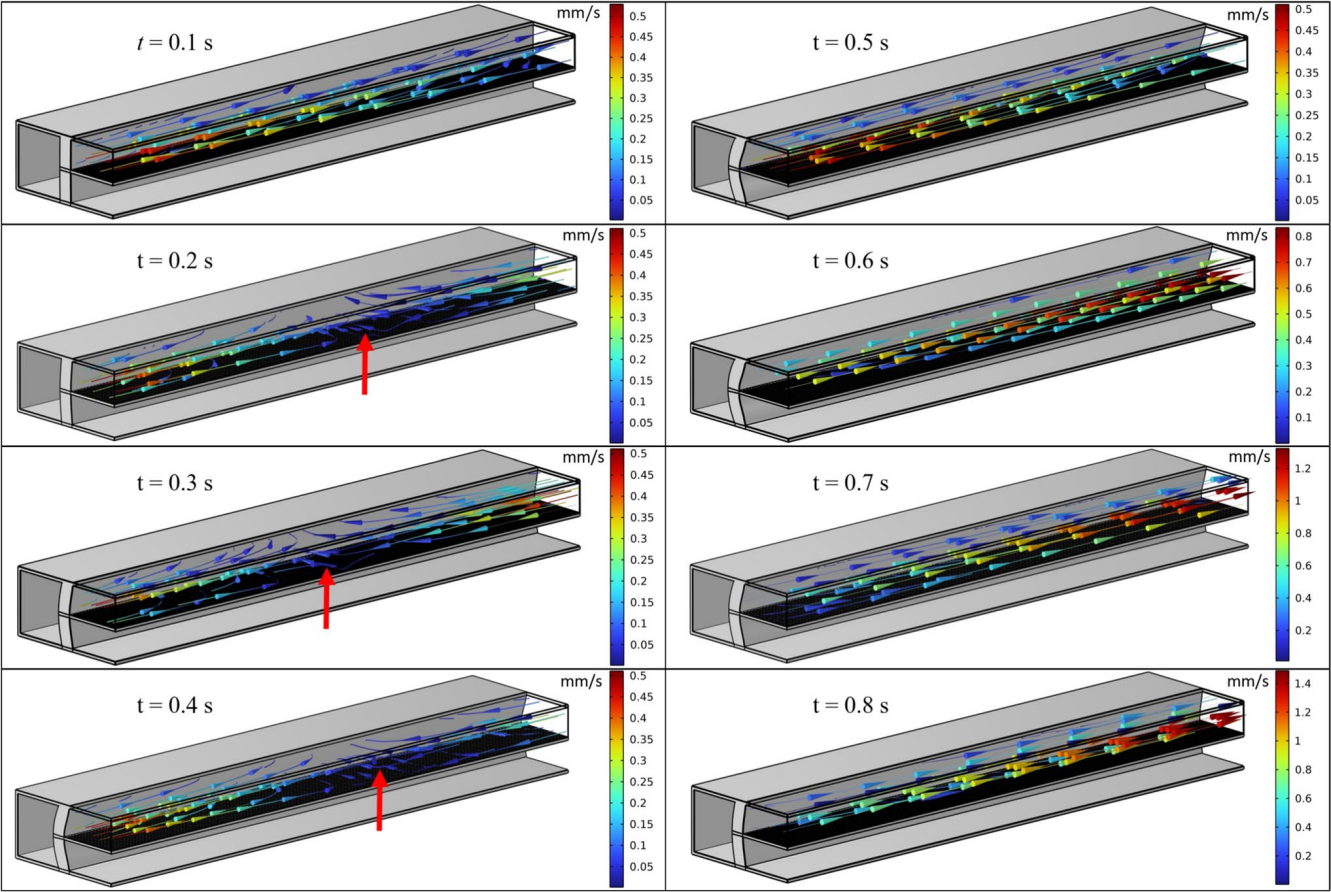
Fluid flow streamlines inside the air channel of the lung-on-a-chip under membrane 15% strain with the frequency of 1 Hz and inflow rate of 0.72 μL/min. Red marks indicate localized flow stagnation regions

As shown in Fig. 8a, higher stretching intensity results in an increase in the likelihood of observing reversed flow and local stagnations within the control volume. This effect is visually highlighted in Fig. 9, where a sequence of snapshots depicts the flow regime during a cycle period with 15% strain intensity and 0.72 μL/min inflow rate. The streamlines in the snapshots portray the direction and magnitude of the flow velocity. In this context, snapshots captured before $t=0.5s$ depict the stretching phase of the membrane, while those taken after $t=0.5s$ correspond to the contraction phase, bringing the membrane back to its normal position. $t=0.5s$ represents a moment of steadiness when maximum tension is applied to the membrane during the cycle. Throughout the stretching phase, when the time derivative of the volume surpasses the inflow rate, localized flow stagnation regions emerge within the microchannel, the location of which is indicated by the red markers in snapshots captured at $t=0.2s$, 0.3 s, and 0.4 s. The position of the local stagnation region relies solely on the volume change rate. This observation suggests that by maintaining a constant volume change rate, the stagnation region remains consistent throughout the stretching phase. This presents an opportunity for precise control over the location of this local stagnation, offering potential utility in delivering aerosol particles to a specific, predetermined region within the microchip.

### Dynamics of nanoparticles in a sinusoidal stretched lung-on-a-chip

**Fig. 10:**
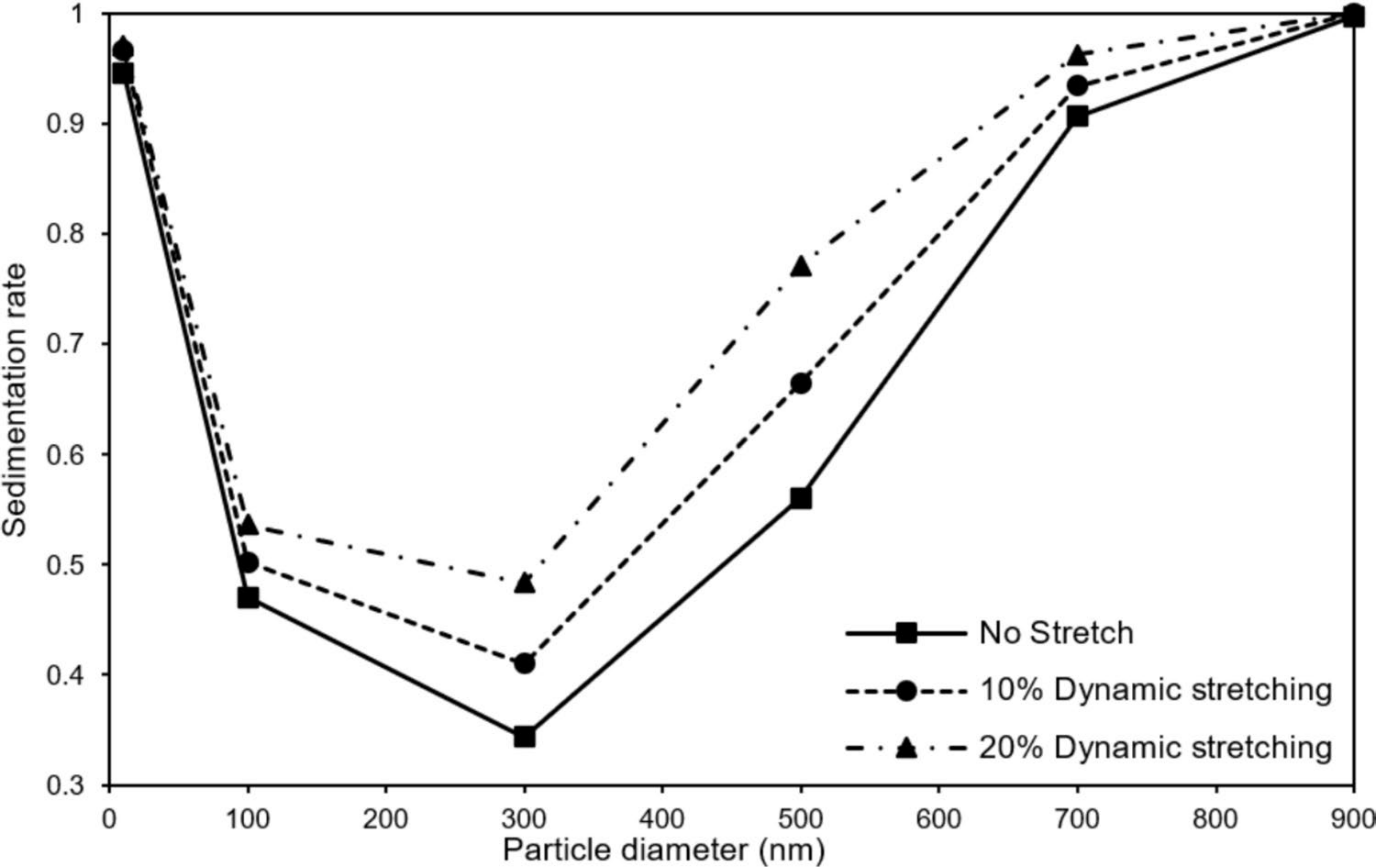
Comparison of sedimentation rate of NPs with different diameters in the air channel with no stretch and under sinusoidal stretch of the 30.5% porous membrane with a frequency of 1 Hz and strains of 10% and 20%. The inflow rate is 0.72 μL/min

This section examines the NPs’ dynamics within the lung-on-a-chip under dynamic stretching of the porous membrane with a high porosity of 30.5%. Accordingly, a sinusoidal pressure pattern is applied to the lateral vacuum channels of the microchip in order to simulate the function of a breathing lung. As demonstrated in the Fluid dynamics inside a sinusoidal stretched lung-on-a-chip section, the overall sedimentation rate rises with the constant membrane stretching, correlating with an increase in the cross-sectional area of the air channel. However, the sedimentation rate changes compared to the state without stretching are small and less than a few percent. On the other hand, the periodic stretching of the membrane with an intensity equivalent to the constant stretching can lead to significant changes in the total sedimentation rate. As discussed in the Dynamics of nanoparticles in a sinusoidal stretched lung-on-a-chip section, the elevation of excitation frequency, particularly during specific time intervals with a higher control volume change rate than the input flow rate, induces a reversal in flow direction at the channel outlet and creates localized fluid stagnation within the channel. Here, the impact of the reverse flow and localized fluid stagnation on NPs dynamics and their overall sedimentation is investigated. Figure 10 presents a comparison of the total sedimentation rate of the NPs with different sizes under varying intensities of periodic stretching. For this, an inlet air channel flow rate of is assumed. To introduce periodic stretching, sinusoidal suction pressure with a frequency of 1 Hz is applied to the side walls of the vacuum channels, and three different conditions (i.e., no stretching, 10% strain, and 20% strain) are considered for the intensity of periodic stretching. As mentioned earlier, the membrane permeability does not significantly affect the NPs'sedimentation rate, and their size is disregarded in this section.

**Fig. 11:**
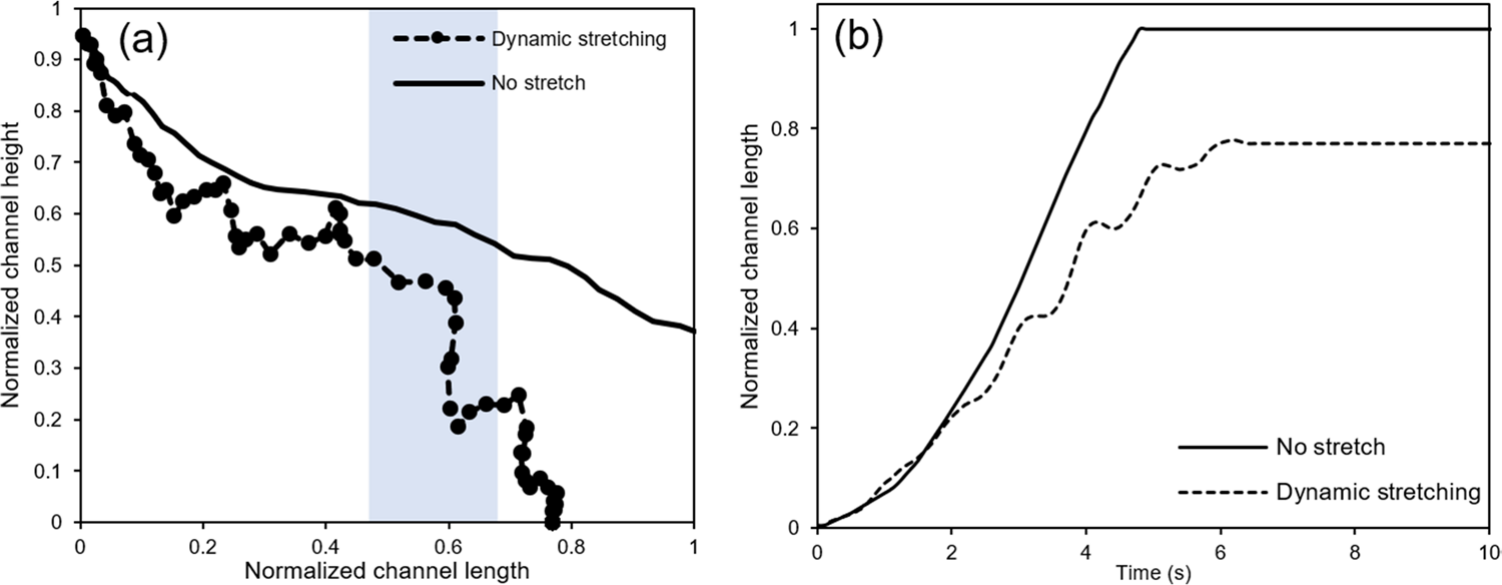
Comparison of the one NP trajectory with a diameter of 500 nm **a**) in the air channel and **b**) its longitudinal motion at different times under sinusoidal stretch with a frequency of 1 Hz and strain of 20%. The inflow rate is 0.72 μL/min

Fig. TEN depicts that the application of periodic stretching leads to an increase in the sedimentation amount, and this increment is further accentuated with an increase in stretching intensity. However, the rate of change is not uniform for NPs with different sizes. The results reveal that NPs with average sizes (e.g., 300 nm and 500 nm) are more susceptible to periodic stretching and induced changes in the flow regime. In line with the results presented in the Dynamics of nanoparticles in a fixed stretched lung-on-a-chip section and Eqs. 9 and 10, the primary factor influencing the dynamic behavior of NPs with average sizes is the drag force, justifying their higher dependence on the flow regime. This is in contrast to smaller and larger NPs, which exhibit different dynamic behaviors, indicating the dominant effects of Brownian and gravitational forces on these NPs, respectively. Moreover, by comparing the results related to the statistical quantities of different particle deposition in Fig. 7, it can be inferred that NPs with average sizes have a more uniform distribution compared to smaller and larger NPs. Hence, it can be concluded that, unlike smaller and larger NPs, which exhibit a greater tendency to sediment near the channel inlet, NPs with average sizes are ordinarily exposed to fluid flow for a longer duration. To elucidate the impact of periodic stretching and localized fluid stagnation on NPs sedimentation rate, the trajectory of an NP with a diameter of 500 nm with uniform initial conditions in two cases: without stretching and with sinusoidal stretching of intensity 20% and frequency 1 Hz is compared in Fig. 11a. In the absence of stretching, the NP follows a relatively smooth path before exiting through the air channel outlet. In contrast, under periodic stretching, the NP’s trajectory exhibits significant oscillations, indicating the influence of changes in flow regimes and localized fluid stagnation on its dynamic motion. Therefore, Fig. 11a serves as an illustration of the effect of periodic stretching on the dynamics of NPs and, consequently, the increased sedimentation rate in NPs with average sizes.

Figure 11b illustrates the longitudinal motion of the NP, providing a direct representation of the impact of sinusoidal stretching on its displacement along the length of the microchannel over a period of 10 s. The results clearly depict the oscillatory motion of the NP under periodic stretching. An important observation is that the time taken for the NP to reach the outlet of the channel without stretching is less than the time required for the same NP to deposit under oscillatory stretching. This result is noteworthy, as it indicates that, despite a uniform flow rate through the microchannel, periodic stretching leads to a longer residence time for NPs in the air channel. Finally, images depicting the motion of the NP with a diameter of 500 nm under periodic membrane stretching are presented alongside flow lines in Fig. S6. The NP’s motion is scrutinized during time intervals between 3.7 and 4.8 s along the spatial domain from the channel inlet, equivalent to the region highlighted in blue in Fig. 11a. The color scheme employed in these images indicates the size and direction of fluid flow. Examining the flow intensity and the flow line directions reveals the occurrence of reverse flow in this section of the air channel and the manifestation of local flow stagnation in the images corresponding to moments 4.2 s, 4.3 s, and 4.4 s. Tracking the NP’s trajectory, it becomes apparent that, simultaneously with the formation of relative stability in fluid flow near the NP, within a relatively short time interval (approximately 0.2 s), the NP undergoes a significant reduction in height. This observation highlights the direct impact of periodic stretching on the sedimentation rate of NPs.

## Conclusion

This study investigated the dynamics of solid NPs within a dual-channel air-medium lung-on-a-chip microdevice, focusing on key influencing factors, including inflow rate, particle size, membrane porosity, and stretching patterns. Accordingly, a comprehensive 3D multi-physics finite element model was developed, integrating solid mechanics, fluid dynamics, and particle tracing to accurately simulate the system's behavior.

The advanced 3D model revealed that a higher stretch of the membrane not only increased the overall NP sedimentation rate but also enhanced membrane porosity, particularly in membranes with initially low porosity. This increased porosity improved NP transfer to the medium channel and influenced deposition patterns in the air channel. Additionally, the effects of sinusoidal membrane stretching on fluid dynamics and NP behavior indicated that exceeding certain stretching frequencies or intensities induced localized flow reversal and temporary stagnation, which can prolong the residence time of NPs inside the air channel and influence their sedimentation pattern. By maintaining a constant volume change rate, the stagnation region remains consistent throughout the stretching phase, providing an opportunity for precise control over its location. This could be leveraged to facilitate the targeted delivery of aerosol particles to specific, predetermined regions within the microchip.

These findings, which underscore the critical role of membrane dynamics and stimulated fluid flow regimes in controlling NPs’ distribution within lung-on-a-chip systems, can effectively contribute to the precise adjustment of particle dosages and chemical formulations to enhance optimal delivery to target cells. This will ultimately improve toxico-pharmacokinetic outcomes derived from the lung-on-chip technology, enabling reliable research in respiratory health and toxicology.

## Supplementary Information

Below is the link to the electronic supplementary material.

Supplementary file1 (DOCX 3005 KB)

## Data Availability

All data generated or analyzed during this study are included in this published article.

## References

[1] Petit P, Maitre A, Persoons R, Bicout DJ. Lung cancer risk assessment for workers exposed to polycyclic aromatic hydrocarbons in various industries. Environ Int. 2019;124:109–20. 10.1016/j.envint.2018.12.058. 30641254 10.1016/j.envint.2018.12.058

[2] Xing YF, Xu YH, Shi MH, Lian YX. The impact of PM2. 5 on the human respiratory system. J ThoracDis. 2016;8:E69. 10.3978/2Fj.issn.2072-1439.2016.01.19. 10.3978/j.issn.2072-1439.2016.01.19 PMC4740125 26904255

[3] Deng Q, Ou C, Chen J, Xiang Y. Particle deposition in tracheobronchial airways of an infant, child and adult. Sci Total Environ. 2018;612:339–46. 10.1016/j.scitotenv.2017.08.240. 28854390 10.1016/j.scitotenv.2017.08.240

[4] Kleinstreuer C, Zhang Z, Donohue JF. Targeted drug-aerosol delivery in the human respiratory system. Annu Rev Biomed Eng. 2008;10:195–220. 10.1146/annurev.bioeng.10.061807.160544. 18412536 10.1146/annurev.bioeng.10.061807.160544

[5] Kleinstreuer C, Zhang Z. Airflow and particle transport in the human respiratory system. Annu Rev Fluid Mech. 2010;42:301–34. 10.1146/annurev-fluid-121108-145453.

[6] Zhao Z, Ukidve A, Kim J, Mitragotri S. Targeting strategies for tissue-specific drug delivery. Cell. 2020;181:151–67. 10.1016/j.cell.2020.02.001. 32243788 10.1016/j.cell.2020.02.001

[7] Hua S, De Matos MBD, Metselaar JM, Storm G. Current trends and challenges in the clinical translation of nanoparticulate nanomedicines: pathways for translational development and commercialization. Front Pharmacol. 2018;9:790. 10.3389/fphar.2018.00790. 30065653 10.3389/fphar.2018.00790 PMC6056679

[8] Newman SP. Drug delivery to the lungs: challenges and opportunities. Ther Deliv. 2017;8:647–61. 10.4155/tde-2017-0037. 28730933 10.4155/tde-2017-0037

[9] Lin KC, Yen CZ, Yang JW, Chung JHY, Chen GY. Airborne toxicological assessment: The potential of lung-on-a-chip as an alternative to animal testing. Mater Today Adv. 2022;14:100216. 10.1016/j.mtadv.2022.100216.

[10] Huh D. A Human Breathing Lung-on-a-Chip. Ann Am Thorac Soc. 2015;12:S42-4. 10.1513/AnnalsATS.201410-442MG. 25830834 10.1513/AnnalsATS.201410-442MG PMC5467107

[11] Huh D, Matthews BD, Mammoto A, Montoya-Zavala M, Hsin HY, Ingber DE. Reconstituting organ-level lung functions on a chip. Science. 2010;328:1662–8. 10.1126/science.1188302. 20576885 10.1126/science.1188302 PMC8335790

[12] Tan J, Guo Q, Tian L, Pei Z, Li D, Wu M, Zhang J, Gao X. Biomimetic lung-on-a-chip to model virus infection and drug evaluation. Eur J Pharm Sci. 2023;180:106329. 10.1016/j.ejps.2022.106329. 36375766 10.1016/j.ejps.2022.106329 PMC9650675

[13] Moura JA, Meldrum K, Doak SH, Clift MJD. Alternative lung cell model systems for toxicology testing strategies: Current knowledge and future outlook. Semin Cell Dev Biol. 2023;147:70–82. 10.1016/j.semcdb.2022.12.006. 36599788 10.1016/j.semcdb.2022.12.006

[14] Sakolish C, Georgescu A, Huh D, Rusyn I. A model of human small airway on a chip for studies of subacute effects of inhalation toxicants. Toxicol Sci. 2022;187:267–78. 10.1093/toxsci/kfac036. 35357501 10.1093/toxsci/kfac036 PMC9154286

[15] Yang S, Chen Z, Cheng Y, Liu T, Yin L, Pu Y, Liang G. Environmental toxicology wars: Organ-on-a-chip for assessing the toxicity of environmental pollutants. Environ Pollut. 2021;268:115861. 10.1016/j.envpol.2020.115861. 33120150 10.1016/j.envpol.2020.115861

[16] Dong J, Qiu Y, Lv H, Yang Y, Zhu Y. Investigation on microparticle transport and deposition mechanics in rhythmically expanding alveolar chip. Micromachines. 2021;12:184. 10.3390/mi12020184. 33673126 10.3390/mi12020184 PMC7917580

[17] Moghadas H, Saidi MS, Kashaninejad N, Nguyen N. Challenge in particle delivery to cells in a microfluidic device. Drug Deliv Transl Res. 2017;8:830–42. 10.1007/s13346-017-0467-3. 10.1007/s13346-017-0467-3 29270808

[18] Rissler J, Gudmundsson A, Nicklasson H, Swietlicki E, Wollmer P, Löndahl J. Deposition efficiency of inhaled particles (15–5000 nm) related to breathing pattern and lung function: an experimental study in healthy children and adults. Part Fibre Toxicol. 2017;14:1–12. 10.1186/s12989-017-0190-8. 28388961 10.1186/s12989-017-0190-8 PMC5385003

[19] Sheidaei Z, Akbarzadeh P, Kashaninejad N. Advances in numerical approaches for microfluidic cell analysis platforms. J Sci Adv Mater Devices. 2020;5(3):295–307. 10.1016/j.jsamd.2020.07.008.

[20] Amin Arefi SM, Tony Yang CW, Sin DD, Feng JJ. Simulation of nanoparticle transport and adsorption in a microfluidic lung-on-a-chip device. Biomicrofluidics. 2020;14:44117. 10.1063/5.0011353. 10.1063/5.0011353 PMC7443171 32849976

[21] Sheidaei Z, Akbarzadeh P, Guiducci C, Kashaninejad N. Prediction of Dispersion Rate of Airborne Nanoparticles in a Gas-Liquid Dual-Microchannel Separated by a Porous Membrane: A Numerical Study. Micromachines. 2022;13:2220. 10.3390/mi13122220. 36557519 10.3390/mi13122220 PMC9785617

[22] Rosmark O, Ibáñez-Fonseca A, Thorsson J, Dellgren G, Hallgren O, Larsson Callerfelt AK, Elowsson L, Westergren-Thorsson G. A tunable physiomimetic stretch system evaluated with precision cut lung slices and recellularized human lung scaffolds. Front Bioeng Biotechnol. 2022;10:995460. 10.3389/fbioe.2022.995460. 36263353 10.3389/fbioe.2022.995460 PMC9574011

[23] Yadav S, Singha P, Nguyen NK, Ooi CH, Kashaninejad N, Nguyen NT. Uniaxial Cyclic Cell Stretching Device for Accelerating Cellular Studies. Micromachines. 2023;14:1537. 10.3390/mi14081537. 37630073 10.3390/mi14081537 PMC10456305

[24] Shourabi AY, Kashaninejad N, Saidi MS. An integrated microfluidic concentration gradient generator for mechanical stimulation and drug delivery. J Sci Adv Mater Devices. 2021;6:280–90. 10.1016/j.jsamd.2021.02.009.

[25] Kamble H, Barton MJ, Jun M, Park S, Nguyen NT. Cell stretching devices as research tools: engineering and biological considerations. Lab Chip. 2016;16:3193–203. 10.1039/C6LC00607H. 27440436 10.1039/c6lc00607h

[26] Shrestha J, RazaviBazaz S, AboulkheyrEs H, Yaghobian Azari D, Thierry B, EbrahimiWarkiani M, Ghadiri M. Lung-on-a-chip: the future of respiratory disease models and pharmacological studies. Crit Rev Biotechnol. 2020;40:213–30. 10.1080/07388551.2019.1710458. 31906727 10.1080/07388551.2019.1710458

[27] Huang Y, Nguyen NT. A polymeric cell stretching device for real-time imaging with optical microscopy. Biomed Microdevices. 2013;15:1043–54. 10.1007/s10544-013-9796-2. 23868118 10.1007/s10544-013-9796-2

[28] Hart KC, Sim JY, Hopcroft MA, Cohen DJ, Tan J, Nelson WJ, Pruitt BL. An easy-to-fabricate cell stretcher reveals density-dependent mechanical regulation of collective cell movements in epithelia. Cell Mol Bioeng. 2021;14:569–81. 10.1007/s12195-021-00689-6. 34900011 10.1007/s12195-021-00689-6 PMC8630312

[29] Cheng YS. Mechanisms of pharmaceutical aerosol deposition in the respiratory tract. AAPS Pharmscitech. 2014;15:630–40. 10.1208/s12249-014-0092-0. 24563174 10.1208/s12249-014-0092-0 PMC4037474

[30] Huh D, Kim HJ, Fraser JP, Shea DE, Khan M, Bahinski A, Hamilton GA, Ingber DE. Microfabrication of human organs-on-chips. Nat Protoc. 2013;8:2135–57. 10.1038/nprot.2013.137. 24113786 10.1038/nprot.2013.137

[31] Hussain M, Madl P, Khan A. Lung deposition predictions of airborne particles and the emergence of contemporary diseases Part-I. Health (Irvine Calif). 2011;2:51–9.

[32] Nikander K, Prince I, Coughlin S, Warren S, Taylor G. Mode of breathing—tidal or slow and deep—through the I-neb Adaptive Aerosol Delivery (AAD) system affects lung deposition of 99mTc-DTPA. J Aerosol Med Pulm Drug Deliv. 2010;23:37. 10.1089/jamp.2009.0786. 10.1089/jamp.2009.0786 PMC3116638 20373908

[33] Frost TS, Estrada V, Jiang L, Zohar Y. Convection-Diffusion molecular transport in a microfluidic bilayer device with a porous membrane. Microfluid Nanofluidics. 2019;23:1–13. 10.1007/s10404-019-2283-1.

[34] Ochs CJ, Kasuya J, Pavesi A, Kamm RD. Oxygen levels in thermoplastic microfluidic devices during cell culture. Lab Chip. 2014;14:459–62. 10.1039/C3LC51160J. 24302467 10.1039/c3lc51160j PMC4305448

[35] Augusto LLX, Gonçalves JAS, Lopes GC. CFD evaluation of the influence of physical mechanisms, particle size, and breathing condition on the deposition of particulates in a triple bifurcation airway. Water, Air, Soil Pollut. 2016;227:56. 10.1007/s11270-016-2753-y.

[36] Domansky K, Leslie DC, McKinney J, Fraser JP, Sliz JD, Hamkins-Indik T, Hamilton GA, Bahinski A, Ingber DE. Clear castable polyurethane elastomer for fabrication of microfluidic devices. Lab Chip. 2013;13:3956–64. 10.1039/c3lc50558h. 23954953 10.1039/c3lc50558h PMC3877836

[37] Shamanskiy A, Simeon B. Mesh moving techniques in fluid-structure interaction: robustness, accumulated distortion and computational efficiency. Comput Mech. 2021;67:583–600. 10.1007/s00466-020-01950-x.

[38] Donea J, Huerta A, Ponthot J, Rodríguez‐Ferran A. Arbitrary L agrangian–E ulerian Methods. Encycl. Comput. Mech., Wiley 2004; 10.1002/0470091355.ecm009

[39] COMSOL AB. Structural Mechanics Module User’s Guide. Sweden, 2018.

[40] Zhang H, Ahmadi G. Aerosol particle transport and deposition in vertical and horizontal turbulent duct flows. J Fluid Mech. 2000;406:55–80. 10.1017/S0022112099007284.

[41] Ariati R, Sales F, Souza A, Lima RA, Ribeiro J. Polydimethylsiloxane Composites Characterization and Its Applications: A Review. Polymers (Basel). 2021;13:4258. 10.3390/polym13234258. 34883762 10.3390/polym13234258 PMC8659928

[42] Si L, Bai H, Rodas M, Cao W, Oh CY, Jiang A, Moller R, Hoagland D, Oishi K, Horiuchi S, Uhl S. A human-airway-on-a-chip for the rapid identification of candidate antiviral therapeutics and prophylactics. Nat Biomed Eng. 2021; 1–15, 10.1038/s41551-021-00718-9 10.1038/s41551-021-00718-9 PMC8387338 33941899

