# Supplementary material for: Dynamics of nanoparticles in a 3D breathing lung-on-a-chip
Source: Drug Deliv Transl Res. 2025 Apr 16;15(12):4693–707. doi: 10.1007/s13346-025-01853-5 (PMC12619719; doi:10.1007/s13346-025-01853-5)
Supplement: Supplementary file 1 — Supplementary file1 (DOCX 3005 KB) [file 13346_2025_1853_MOESM1_ESM.docx]

**Supplementary Information**

**Dynamics of Nanoparticles in a 3D Breathing Lung-on-a-chip**

*Zohreh Sheidaei, Pooria Akbarzadeh * and Navid Kashaninejad **

Z. Sheidaei

Faculty of Mechanical Engineering, Shahrood University of Technology, Shahrood, Iran.

Environmental Engineering Institute, Swiss Federal Institute of Technology Lausanne, Lausanne, Switzerland.

P. Akbarzadeh

Faculty of Mechanical Engineering, Shahrood University of Technology, Shahrood, Iran.

Institute of Fluid Mechanics and Heat Transfer, Johannes Kepler University Linz, Linz, Austria.

akbarzad@ut.ac.ir, p.akbarzadeh@jku.at, Tel.: +43-67764781760

N. Kashaninejad

Queensland Micro- and Nanotechnology Centre, Nathan Campus, Griffith University,170 Kessels Road, Brisbane, QLD 4111, Australia.

n.kashaninejad@griffith.edu.au, +61-737355391

1. **Experimental setup**

The PDMS microchannels were fabricated using conventional photolithography and soft lithography procedures in a clean room environment [1]. Both particle sizes (1µm and 10µm in diameter) were accommodated within channels of the same width (2000 µm) and length (15000 µm), though the channel’s height was adjusted to 300 µm for larger particles and 100 µm for smaller ones, aiding their navigation through the channel. A PHD ULTRA™ syringe pumps (Harvard Apparatus, Holliston, MA, USA) was used to inject the microparticles into the microchannel. The particle distribution was monitored using an optical microscope (Leica DM2500M). Due to the microscope’s limited field of view, a series of images from consecutive segments of the channel substrate was captured and then merged into one comprehensive image of the entire channel length.

To analysis the mechanical strain of the lung-on-a-chip membrane against vacuum pressure, the lateral vacuum channels were connected to a pressure controller pressure system (OB1, Elveflow, Paris, France) equipped with a vacuum pump (Figure S1). The lung-on-a-chip used in this study was generously provided by the Laboratory of Microbiology and Microtechnology at the Swiss Federal Technology Institute of Lausanne.


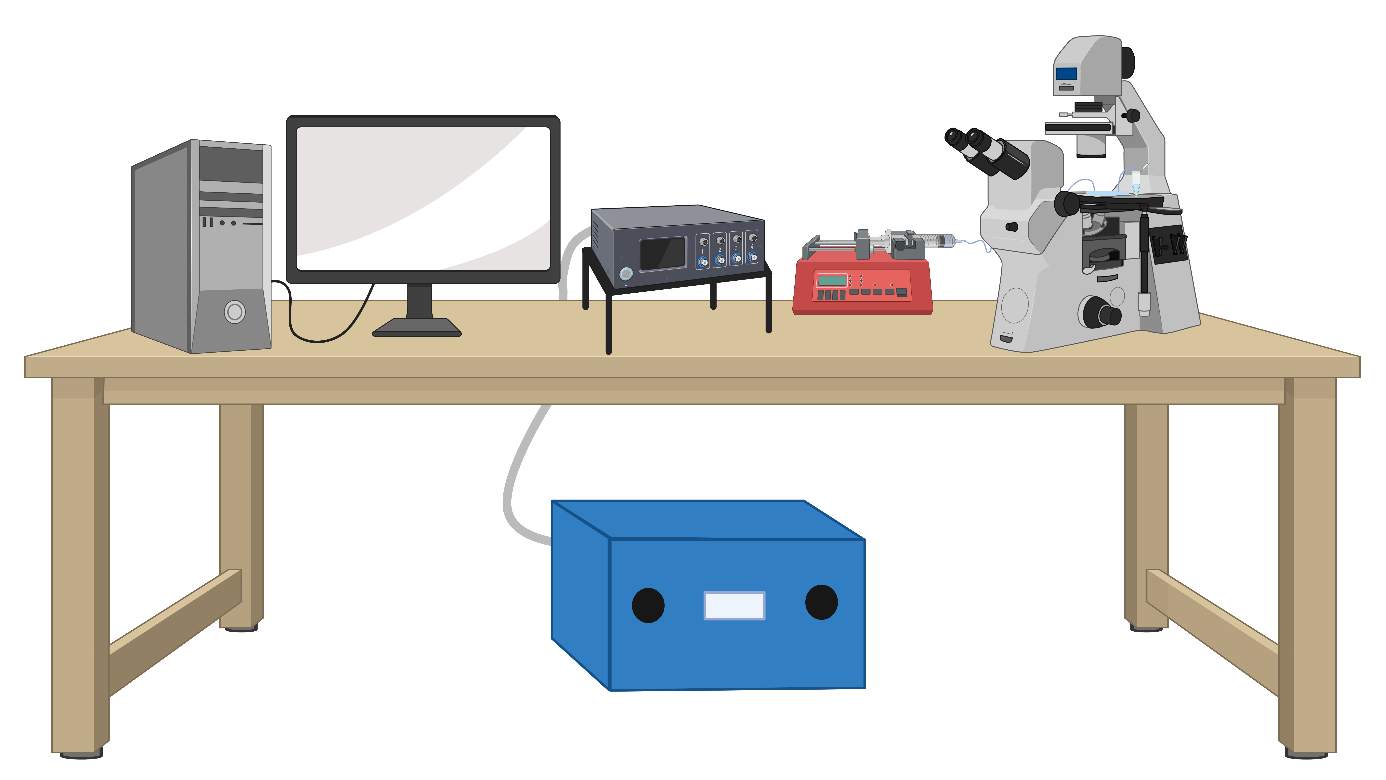


Microfluidic chip

Vacuum pump

Pressure controller

Syringe pump

**Fig. S1** schematic picture of the experimental setup employed for on-chip experiments.

1. **Comparison of nanoparticle dynamics in 2D and 3D models**

At the outset, comparing the current 3D simulation with an earlier 2D model [2] is critical to identify the strengths and limitations of the 2D approach and highlight the need for 3D modeling. The 2D model inadequately represents the realistic configuration of membrane pores, leading to inaccurate porosity rates and variations in deposition and transfer rates compared to the more comprehensive 3D model. To address this, the sedimentation rate—a combined measure of deposition and transfer rates—is analyzed for both 2D and 3D models, as shown in Fig. S2. This comparison aims to provide a clearer understanding of the particle dynamics that influence sedimentation rates.

The observed higher sedimentation rates for smaller particles in the 3D model can primarily be attributed to Brownian diffusion, which, according to the Stokes-Einstein relation [3,4], is the predominant mechanism for the deposition of smaller particles. Conversely, gravitational acceleration plays a more significant role for larger particles, as indicated by Equations 9 and 10, while the influence of Brownian motion decreases. This distinction underscores the necessity and advantage of 3D modeling in capturing the nuanced behaviors of nanoparticles within the lung-on-a-chip’s microenvironment.

Furthermore, Fig. S2 illustrates a notable decrease in sedimentation rates as the flow rate increases, resulting in a higher proportion of NPs exiting the microdevice via the air channel outlet. This trend, consistent with the data presented in Fig. S2, underscores the 3D model’s accuracy. However, minor differences can largely be ascribed to variations in hydrodynamic flow regimes. Unlike the 2D model, which presupposes uniform flow velocity across the microchip’s width, the 3D model predicts a parabolic flow profile. This is due to the moderate aspect ratio of the air channel (w/h = 4, as shown in Fig. 1), which deviates from the uniform flow assumption inherent in the 2D approach.


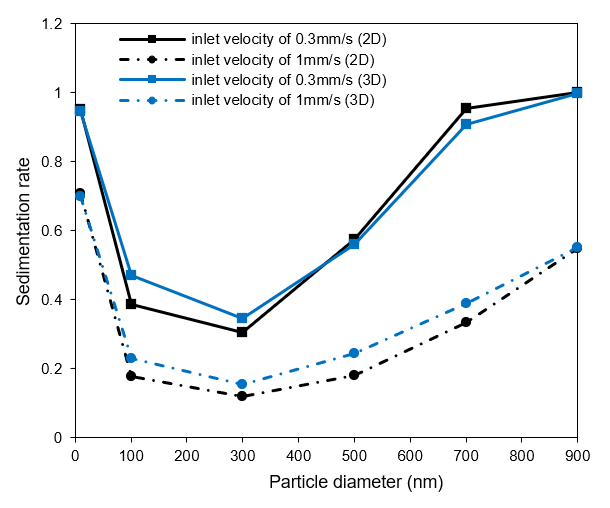


**Fig. S2** Comparison of NPs sedimentation rate on the air channel substrate in 2D and 3D models under two different inflow velocities of $0.3 mm/s$ and $1 mm/s$

Fig. S3 showcases dimensionless distribution indexes for the total sedimented NPs, encompassing those deposited on the air channel substrate and those that have traversed the pores into the medium channel. Specifically, Fig. S3a illustrates the average position of sedimented NPs along the channel, normalized by the channel length, while Fig. S3b displays the normalized standard deviation of NP distribution across the channel. The reference red solid lines in the figures denote an ideal uniform deposition scenario, characterized by a normalized mean of 0.5 and a standard deviation of 1/√12, as per the statistical theory [5].

The impact of membrane porosity on the statistical characteristics of NPs distribution is noted to be negligible in this context. In the numerical simulation, NPs passing through the pores are considered immobile upon reaching the air channel substrate level before pore entry to facilitate further statistical analysis. Fig. S3 highlights the effects of Brownian, drag, and gravity forces on the distribution of NPs, demonstrating similarities between the results of 2D and 3D models as detailed in Ref. [2]. The discussion here focuses more on elucidating the differences observed between the two models.


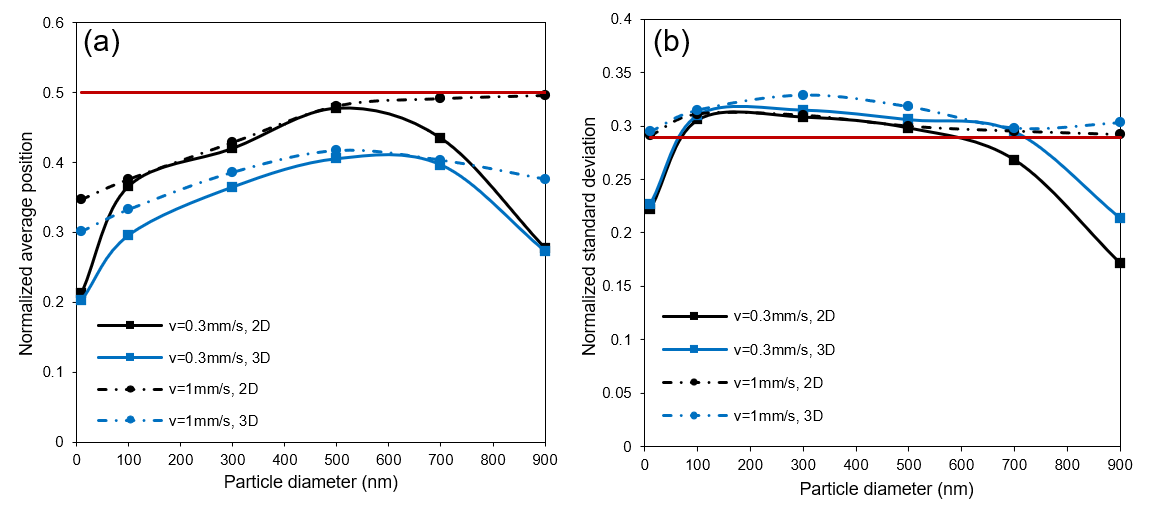


**Fig. S3** Comparison of normalized (a) average position and (b) standard deviation of deposited NPs on the substrate of the air channel in the 2D and 3D models under two different fluid velocities of 0.3 mm/s and 1 mm/s. The solid red line corresponds with an ideal uniform distribution

From Figs. S2 and S3, it is clear that the parabolic flow regime in the 3D model impacts nanoparticles differently at various inflow velocities. Therefore, three examples are provided to clarify how the flow regime change in 2D and 3D models leads to deviations in the obtained results.

**Example 1**: 900nm particles – inlet flow velocity of o.3 mm/s

The sedimentation rate equals 1 in Fig. S2 for the 2D simulation, indicating that all nanoparticles with a diameter of 900nm tend to deposit on the air channel substrate or pass through the pores into the medium channel. Additionally, the average position and standard deviation values in Fig. S3 suggest that most NPs deposit near the air channel’s inlet. Under the parabolic flow regime of the 3D model, even the front NPs cannot reach the outlet to exit the air channel. Thus, the sedimentation rate is expected to mirror that of the 2D model, as confirmed in Fig. S2. With no NPs exiting the channel, the average position of the NPs is predicted to align with the 2D model, as illustrated in Fig. S3a. This is because the movement of particles in the central channel area offsets the slower progression at the sides due to the parabolic flow shape. However, the flow velocity variance between the center and sides of the channel results in a broader distribution pattern in the 3D model, as seen in Fig. S3b.

For a direct comparison of the 3D model results with those from the 2D model, Fig. S4 presents a series of snapshots showing the sedimentation patterns of NPs. A shallow channel model simulates the 2D model for this comparison. Red curves represent each case’s flow regime at the air channel entrance. Fig. S4a compares the particle distribution in 3D and shallow channel models for this example case.

**Example 2:** 10 nm particles – inlet flow velocity of 1 mm/s

As shown in Supplementary Fig. 2, the sedimentation rate for particles of 10 nm diameter is approximately 0.71 under an inflow velocity of $1 mm/s$ in the 2D model. This indicates that around 30% of the particles pass through the air channel and exit via the outlet without sedimentation. When subjected to the parabolic flow regime in the 3D model, particles situated at the forefront in the central region of the channel exhibit a slightly advanced progression, leading them to exit the channel through the outlet. Simultaneously, the lower flow rate along the channel’s sides induces regression in the NPs, preventing a portion of them from leaving the channel. This phenomenon is clearly illustrated in Fig. S4b, where red points at the outlet signify exiting particles. While the standard deviation of sedimented particles remains consistent, as depicted in Fig. S3b, the regression of particles along the channel’s sides causes a reduction in their average position, as illustrated in Supplementary Fig. 3b.

**Example 3:** 500 nm particles – inlet flow velocity of 0.3 mm/s

Fig. S3c compares the particle distribution between the 3D and shallow channel models for NPs with a diameter of 500 nm, subjected to an inflow velocity of 0.3 mm/s. It reveals a reduction in the concentration of sedimented NPs along the sides of the channel when approaching the outlet for the 3D model. It could be observed that fewer particles tend to exit from the sides, while a greater number of particles leave from the central regions, in comparison to the shallow model. Here, the count of additional particles leaving the channel from the central region of the 3D model is comparable with those leaving from the sides of the shallow channel. Consequently, the sedimentation rate remains almost constant, as illustrated in Fig. S2. However, a noteworthy difference in the distribution pattern is evident with the average particle position shifting closer to the channel inlet in the 3D model, whereas the standard deviation exhibits negligible alteration, as depicted in Fig. S3.

**
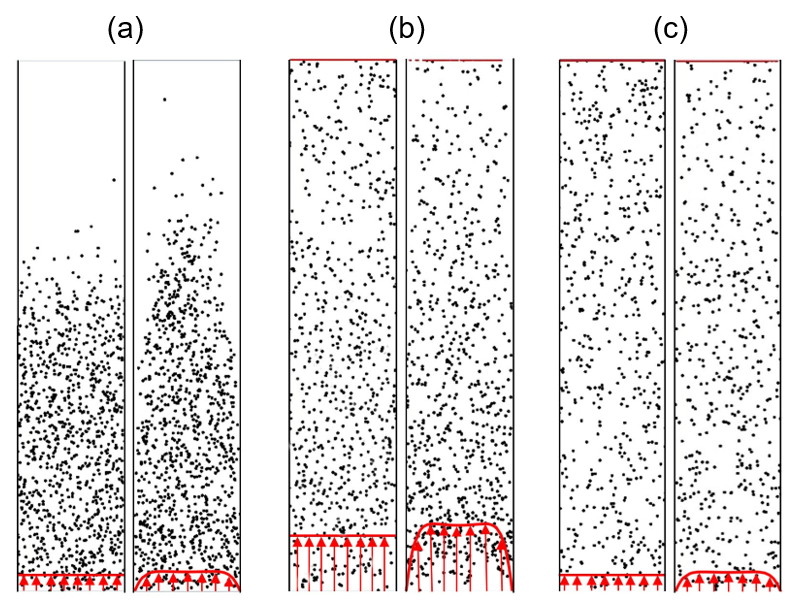
**

**Fig. S4** Comparison of deposited NPs’ distribution in a shallow channel (left columns) and current 3D model (right columns); a) $d_{p}=900nm$, inflow velocity of o.3 mm/s; b) $d_{p}=10nm$, inflow velocity of 1 mm/s; c) $d_{p}=500nm$, inflow velocity of 0.3 mm/s. Red curves at the entrance depict the velocity profile, while red points at the outlet signify exiting NPs. The pores are eliminated from the substrate to enhance the visibility of the NPs distribution

Considering the significantly lower computational cost associated with 2D modelling, it proves advantageous in furnishing valuable insights into particle dynamics and the impact of various parameters such as particle size and inlet velocity. However, 2D modeling limitations become apparent in accurately depicting the actual membrane porosity that results in distinct variations in deposition and transfer rates. Furthermore, it assumes a uniform flow velocity across the microchip width, a simplification that contrasts with the more representative 3D model that suggests a realistic profile for the flow regime and enhances the accuracy of particle tracing.

1. **Fluid pressure gradient inside a sinusoidal stretched lung-on-a-chip**

Fig. S5 illustrates the average normalized pressure gradient along the channel length throughout a stretching cycle period. The pressure is normalized with respect to the kinetic energy per unit volume of the inlet flow. The results consist of four cases for two distinct excitation frequencies and two different inflow rates. According to the atmospheric pressure boundary condition at the channel outlet, the relative pressure remains consistently at zero regardless of operating conditions. Following the Navier-Stokes momentum equation, when the volume control size remains constant, the pressure gradient exhibits a linear profile along the channel length (e.g., at $\bar{t}=0$ and $\bar{t}=0.5$ in Fig. S5). However, during the stretching and contraction phases, the pressure gradient takes on the shape of a second-order convex or concave curve, respectively. When the time derivative of the volume surpasses the inflow rate in the stretching phase, a local minimum in the pressure gradient emerges that corresponds to the presence of local stagnation regions. This is evident, for example, in Fig. S5a at $\bar{t}=0.25$, located at a normalized distance of ~0.4 under 20% strain and ~0.65 under 10% strain. This observation aligns with previous findings and could be extracted from Fig. S4a too, where zero flow rate indicates the stagnation region.

*
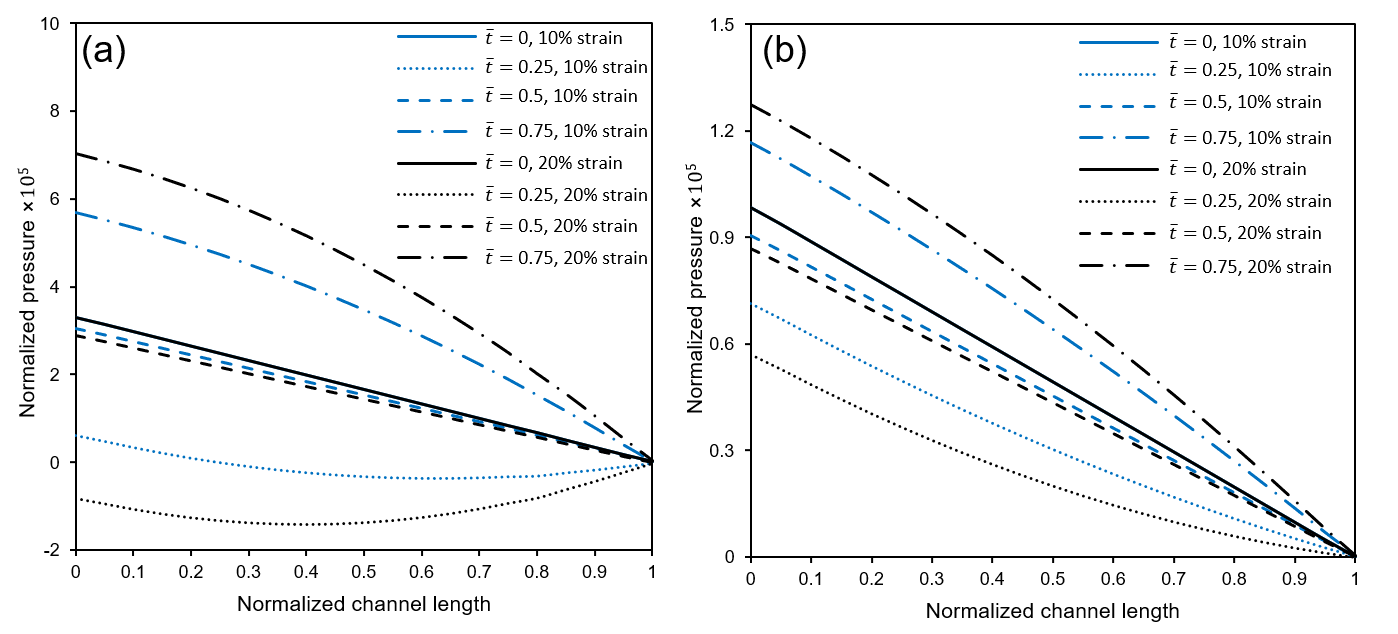

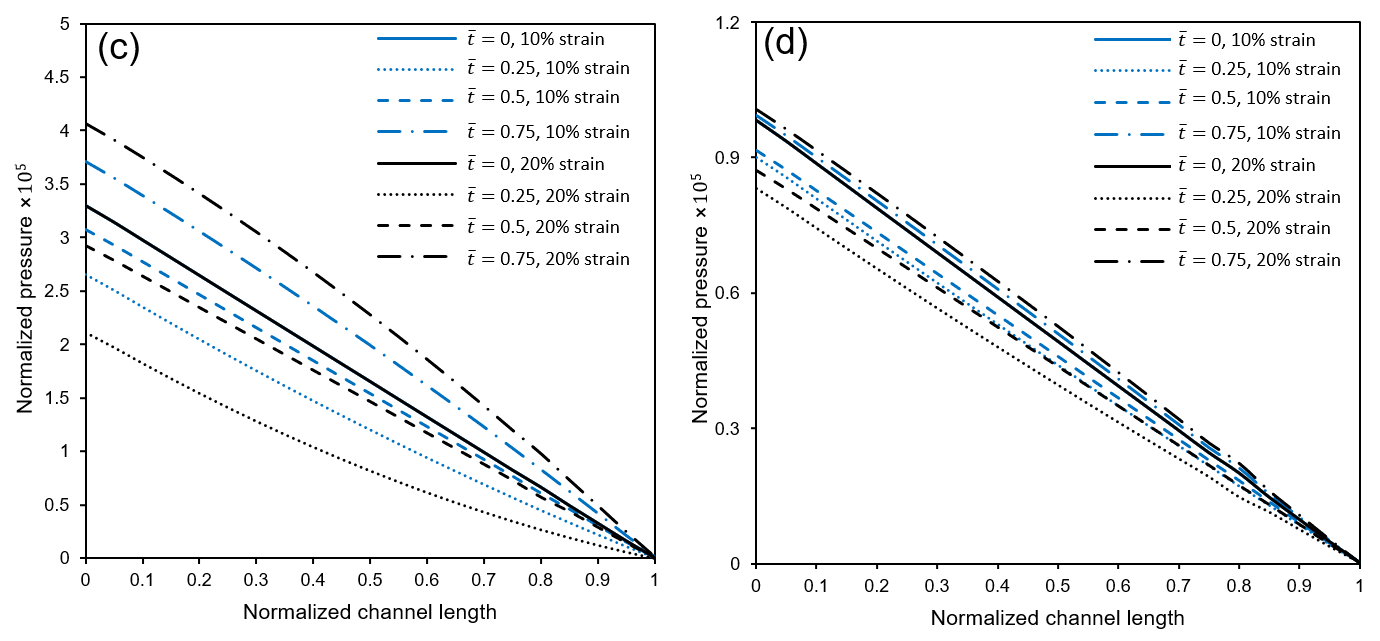
*

**Fig. S5** Average normalized pressure along the air channel under two different membrane stretching intensities at various times when a) stretching frequency is 1 Hz and inflow rate is $0.72 {\mu L}/\min$, b) stretching frequency is 1 Hz and inflow rate is $2.4 {\mu L}/\min$, c) stretching frequency is 0.25 Hz and inflow rate is $0.72 {\mu L}/\min$, and d) stretching frequency is 0.25 Hz and inflow rate is $2.4 {\mu L}/\min$. Average pressure and distance are respectively normalized with respect to the kinetic energy per unit volume of the inlet flow and channel length, while time is normalized concerning the stretching cycle period

1. **Nanoparticle trajectory inside a sinusoidal stretched lung-on-a-chip**

To further illustrate the influence of periodic stretching on particle dynamics, Fig. S6 shows a sequence of flow field snapshots and the trajectory of a 500 nm nanoparticle over time (from 3.7 s to 4.8 s) under sinusoidal stretching (1 Hz, 20% strain). These snapshots clearly highlight the emergence of local flow reversal and stagnation, particularly between 4.2 s and 4.4 s, where the particle undergoes a rapid vertical displacement due to the transient reduction in flow velocity

**
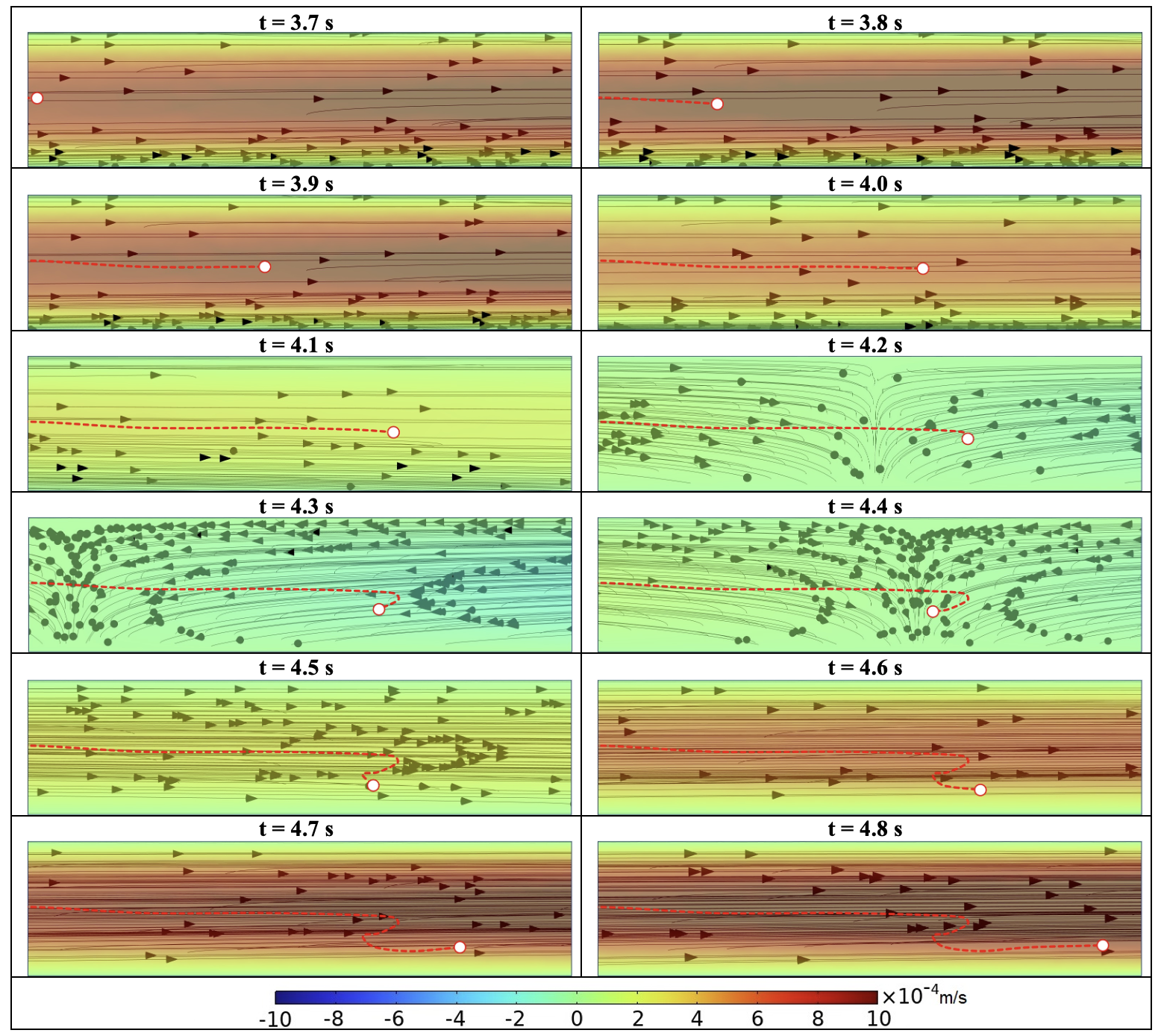
 Fig. S6** Snapshots of the one NP trajectory with a diameter of 500nm in the air channel where the local stagnation due to the reverse flow occurs under sinusoidal stretch with a frequency of 1Hz and strain of 20%. The inflow rate is $0.72 {\mu L}/\min$

[1] Huang Y, Nguyen NT. A polymeric cell stretching device for real-time imaging with optical microscopy.

Biomed. Microdevices **2013**; 15, 1043–54; <http://dx.doi.org/10.1007/s10544-013-9796-2>

[2] Sheidaei Z, Akbarzadeh P, Guiducci C, Kashaninejad N. Prediction of Dispersion Rate of Airborne Nanoparticles in a Gas-Liquid Dual-Microchannel Separated by a Porous Membrane: A Numerical Study. Micromachines **2022**; 13, 2220; <https://doi.org/10.3390/mi13122220>

[3] Friedlander SK. Smoke, Dust, and Haze: Fundamentals of aerosol dynamics. Oxford University Press, New York 2000.

[4] Kohli R, Mittal KL. Developments in surface contamination and cleaning-Vol 2: Particle deposition, control and removal. William Andrew; 2009.

[5] Hayter AJ. Probability and statistics for engineers and scientists. 3rd ed. Duxbury : Thomson Brooks/Cole; **2002**.
